# *ExOrthist*: a tool to infer exon orthologies at any evolutionary distance

**DOI:** 10.1186/s13059-021-02441-9

**Published:** 2021-08-20

**Authors:** Yamile Márquez, Federica Mantica, Luca Cozzuto, Demian Burguera, Antonio Hermoso-Pulido, Julia Ponomarenko, Scott W. Roy, Manuel Irimia

**Affiliations:** 1 Centre for Genomic Regulation, Barcelona Institute of Science and Technology, Dr. Aiguader, 88, 08003 Barcelona, Spain; 2 Universitat Pompeu Fabra, Barcelona, Spain; 3 Department of Zoology, Charles University, Vinicna 7, 12844 Prague, Czech Republic; 4 San Francisco State University, 1600 Holloway Ave, San Francisco, CA 94132 USA; 5 ICREA, Barcelona, Spain

**Keywords:** Alternative splicing, Orthology, Paralogy, Intron-exon structures

## Abstract

Several bioinformatic tools have been developed for genome-wide identification of orthologous and paralogous genes. However, no corresponding tool allows the detection of exon homology relationships. Here, we present *ExOrthist*, a fully reproducible *Nextflow*-based software enabling inference of exon homologs and orthogroups, visualization of evolution of exon-intron structures, and assessment of conservation of alternative splicing patterns. *ExOrthist* evaluates exon sequence conservation and considers the surrounding exon-intron context to derive genome-wide multi-species exon homologies at any evolutionary distance. We demonstrate its use in different evolutionary scenarios: whole genome duplication in frogs and convergence of *Nova*-regulated splicing networks (https://github.com/biocorecrg/ExOrthist).

**Supplementary Information:**

The online version contains supplementary material available at 10.1186/s13059-021-02441-9.

## Background

**Fig. 1:**
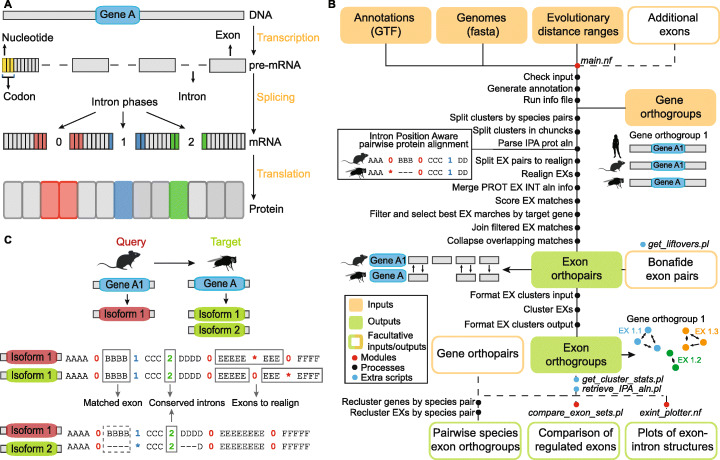
Molecular background and overview *of ExOrthist*. **a** Schematic representation of the exon-intron structure in eukaryotic protein-coding genes and of the molecular events necessary to generate a protein. The DNA is transcribed into pre-mRNA, from which introns are removed through the process of splicing. The exons are joined in the mature mRNA, where each adjacent group of three nucleotides (codon) encodes a different amino acid. Depending on the codon status at the exon-exon junctions, the removed introns are classified as being in phase 0 (complete codons on both sides of the junction) or phase 1 or 2 (split codons across the junction). The mature mRNA is then translated into a protein. **b** Overview of the *ExOrthist* pipeline, including inputs, outputs, processes, additional functionalities, and modules. **c** Schematic representation of species pairwise IPA alignments generated by *ExOrthist*. Given a query and a target gene, *ExOrthist* aligns all query gene non-redundant protein isoforms against all target gene non-redundant protein isoforms and introduces the position of each intron respect to the protein-coding sequence as a 0, 1, or 2, depending on its phase. The figure highlights some common scenarios in IPA alignments: exon matches detected in only one of the aligned isoforms, introns with conserved phases, and query exons matching multiple target exons (which will be specifically realigned)

One of the most fascinating innovations of eukaryotic organisms was the evolution of gene structures composed of exons—pre-mRNA sequences joined and translated into proteins—and interspersed introns—removed from the pre-mRNA through the process of splicing (Fig. 1A). Such innovation greatly increased the evolutionary potential of eukaryotic genomes. First of all, although rarely, changes in the exon-intron structure can modify the function of conserved genes (e.g., [1, 2]). More commonly, many eukaryotic genes acquire the ability to differentially combine their exons and introns into multiple isoforms through the process of alternative splicing. In particular, the inclusion or removal of an entire exon from an isoform (exon skipping) is the most common type of alternative splicing in metazoans and the main contributor to alternative splicing-driven proteome diversification [3]. Exon skipping has been shown to be fast-evolving [4–6], although some cassette exons are exceptionally conserved at the genomic and regulatory level [7]. Furthermore, in several cases, novel alternative splicing events have been linked to organismal innovations [8, 9]. Thus, exon-intron structure and alternative splicing patterns are two relevant intragenic features that have the potential to mediate functional diversification within conserved genes. However, how to link such features to function remains an open challenge [10, 11].

Comparative genomics has been an important tool to link whole genes to functions by inferring gene presence/absence in different species through the identification of evolutionarily related gene orthologs (originated by a speciation event) and paralogs (derived from a duplication event). While orthologs usually maintain the ancestral function, paralogs are free to mutate and evolve new functions, as long as the ancestral role is preserved [12, 13]. Remarkably, although multiple bioinformatic tools have been developed to identify homology relationships at the gene level [14–18], and some are emerging to investigate the conservation of transcript isoforms and/or splicing patterns [19–21], no corresponding tool currently exists to infer homology relationships at the exon level, particularly over deep evolutionary timescales. Nonetheless, even in the absence of general-purpose tools, a variety of studies have underscored the importance of this approach. First, in much the same manner as origins of novel genes have been linked to evolution of new molecular functions, de novo origins of alternatively spliced exons have been associated with evolution of novel gene functions [7, 8]. Second, in the same way as gene paralogs frequently evolve new functions, duplicated paralogous exons have been shown to provide novel functional properties to the host gene [22, 23]. Moreover, even in cases in which new functions do not emerge, mutually exclusive splicing of duplicated exon pairs can lead to the specialization of alternative transcripts for distinct pre-existing functions, mirroring the impact of whole gene duplication [24].

General tools for assigning and studying orthologous and paralogous exons are thus crucial to properly investigate the evolution of gene structures and alternative splicing. For a few model organisms, it is possible to confidently infer pairs of orthologs at short evolutionary distances (e.g., within mammals or within fruitflies) using *liftOver* [25]. Besides the restriction to short evolutionary distances, this approach relies on the availability of precomputed UCSC pairwise chain alignments and can be applied only to one species pair at a time (i.e., multi-species orthogroups cannot be inferred). Moreover, *liftOver* derives exon orthopairs exclusively based on interpolation of the target exon sequence within whole genome alignments, which has two main limitations. First, it does not take into account the preservation of the two neighboring intron positions, whose ancestrality is a requisite for "full" homology of a pair of exons. Second, it does not assess the conservation of the upstream and downstream exons, which helps to further support homology of the neighboring intron positions.

To overcome these limitations and fulfill the major need for a software for exon homology inference, we have developed *ExOrthist*, which uses Intron Position Aware (IPA) protein alignments to infer species pairwise exon homologs and multi-species orthogroups, with substantial flexibility to allow fine tuning according to evolutionary distance. *ExOrthist* has three modules. The *main* module is a *Nextflow*-based pipeline [26] that infers exon homologies using an innovative approach, namely evaluating three features: conservation of the neighboring intron positions (with respect to the protein-coding sequence), sequence and length conservation of the query exons, and sequence conservation of the upstream/downstream exons. Second, the *exint_plotter* module is also a *Nextflow*-based pipeline that allows the visualization of evolutionary conservation and of changes in exon-intron structure among gene homologs of interest. Third, the *compare_exon_sets* module assesses the conservation of alternative splicing patterns between pairs of species. Here, we provide the first description of *ExOrthist.* We first give an overview of the pipeline (schematized in Fig. 1B), describe how we selected the default conservation cut-offs for three evolutionary distance ranges, and compare *ExOrthist* and *liftOver* performances on a set of closely related mammalian species. Then, we illustrate the use of *ExOrthist* to (i) reconstruct paralogous relationships and asymmetric evolution after a whole genome duplication event in the *Xenopus* lineage; and (ii) compare *Nova*-regulated exons in mouse and fruitfly, unveiling a substantial degree of convergent evolution of *Nova* regulation, which has independently targeted orthologous genes in both lineages. All *ExOrthist* modules are freely available at https://github.com/biocorecrg/ExOrthist.

## Results

### Overview of *ExOrthist main* module

The *ExOrthist main* module infers exon homologies within a set of species for which annotation files (in GTF format) and genome files (in fasta format) are provided. Moreover, it is possible to provide additional non-annotated exons identified from RNA-seq data by any splicing quantifier (e.g., [27–31]). *ExOrthist* derives exon homologies at two different levels: (i) exon orthopairs, representing pairs of homologous exons from two different species, and (ii) exon orthogroups, which include all the exons sharing a common ancestor (and thus both orthologs and paralogs) for multiple species. As such, orthopairs and orthogroups provide complementary information that is necessary for the complete reconstruction of exon evolutionary relationships. Since, by definition, exon orthopairs/orthogroups are derived from evolutionarily related genes, *ExOrthist* also requires as input a set of gene orthogroups for the species of interest (e.g., generated by *Orthofinder* [17], *Broccoli* [18], or similar tools). While *ExOrthist* is designed to infer genome-wide exon homologies, it can also be restricted to one/few genes of interest by providing only a subset of gene orthogroups as input. *ExOrthist* utilizes a set of conservation cut-offs (number of conserved intron positions, minimum exon sequence similarity, minimum exon length ratio, minimum global protein similarity; see below), which can be differentially fine-tuned for three evolutionary distance ranges (short, medium, and long). Thus, the evolutionary distance range between each pair of considered species also has to be specified (see online README for further details on the input).

Once launched, *ExOrthist* extracts exon/intron-related information from the annotation files of all the species considered, and then it performs the subsequent steps for each pair of species. First, it generates IPA alignments for all non-redundant protein isoforms belonging to the same gene orthogroup for each species pair (species1–species2) (Fig. 1C). This all-vs-all isoform comparison is necessary because a selection of representative isoforms might leave some exon orthologs undetected simply because they are not included in any of the selected protein isoforms. Considering species1 as query and species2 as target (and vice versa), *ExOrthist* then parses all pairwise isoform alignments. For each query exon, it first identifies the best matching exon in each target isoform based on the global protein alignment and sequence similarity. Next, for each best exon match per target gene isoform, *ExOrthist* evaluates its potential homology with the query exon based on the conservation cut-offs provided (see below). If the comparison between the query and target exon passes all the cut-offs, they are considered as putative homologs. Then, to select the best exon match for each target gene among all putative homologs (from the different isoforms), a global score [0-1] is calculated as the sum of five partial scores reflecting the conservation of the different features of the exon-intron context that were separately evaluated to assess homology: (1, 2) conservation of the immediately upstream and downstream intron positions and phases, (3) conservation of the query exon sequence and (4, 5) conservation of the immediately upstream and downstream exon sequences. The exon match (i.e., query exon - target isoform) with the highest global score among those passing all conservation cut-offs is selected as best match for that target gene, and the matching query and target exons are thereafter considered an orthopair. Importantly, while the *ExOrthist* logic requires a query exon to match a unique exon in the target gene, each exon in the target gene can potentially be matched by multiple query exons: this setting captures cases of in-tandem exon duplication within the same gene while preserving the information about which duplicated exon is more similar to the pro-ortholog in the other species. Furthermore, the user has the possibility to provide custom *bona fide* exon orthopairs, which will be directly incorporated into the generation of the exon orthogroups. This can be obtained from manual curation (e.g., through literature searches), or in a genome-wide manner using the *liftOver* tool. For this purpose, *ExOrthist* provides an additional script to perform *liftOver* between any pair of species for which UCSC *liftOver* files are available.

After deriving species pairwise exon homology relationships, *ExOrthist* infers multi-species exon orthogroups. The exon orthopairs for all the species pairs are joined and translated into a directed graph using the R *igraph* package [32], where an edge-betweenness algorithm selects the optimal topology, i.e., with high intra-community but low inter-community connections. The exon communities identified in the optimal topology correspond to the exon orthogroups returned by *ExOrthist*. Finally, *ExOrthist* offers the additional functionality to generate orthogroups restricted to two species when species pairwise gene orthopairs are provided as extra input.

### *ExOrthist* calibration and benchmarking

*ExOrthist* allows the user to differentially specify a set of conservation cut-offs across three evolutionary distance ranges (short, medium, long): (a) minimum global protein similarity (“prot_sim”); (b) minimum number of conserved intron positions (0, 1 or 2; “int_num”); (c) minimum exon sequence similarity (“ex_seq”); and (d) minimum exon length ratio (shortest_exon/longest_exon; “ex_len”). This flexibility in the definition of cut-offs is particularly relevant, since the use of fixed conservation parameters across different evolutionary distances would result in noisy homology calls for closely related species and incomplete homolog detections for more distant species.

Minimum global protein similarity, or cut-off (a), is the minimum sequence similarity over the entire pairwise protein alignment for a pair of protein isoforms to be considered for further comparisons. While it has no effect on the downstream analyses, it avoids processing spurious gene orthologs or poorly annotated isoforms. The minimum number of conserved introns, or cut-off (b), refers to the requirement for both, one or none of the neighboring intron positions to be conserved, and hence inferred to be ancestral (Fig. 1A). A pair of introns from two genes is considered to be in conserved positions if they have the same phase (i.e., interrupt a codon in the same nucleotide position; Fig. 1A) and are in a comparable location of the protein alignment. In particular, the maximum distance allowed between the two conserved intron positions in the protein alignment depends on the local protein sequence similarity and percentage of unaligned residues (see “Methods”) [33]. Other features, such as intronic length or intronic sequence similarity, are not taken into consideration to assess conservation of intron positions. In this study, we consider homologous internal exons only those for which both neighboring intron positions are conserved; therefore, this cut-off is always set to 2 in all the following analyses. However, by lowering the number of required conserved introns to one, internal exons sharing a single intron position can also be accommodated in the final orthologies.

**Fig. 2:**
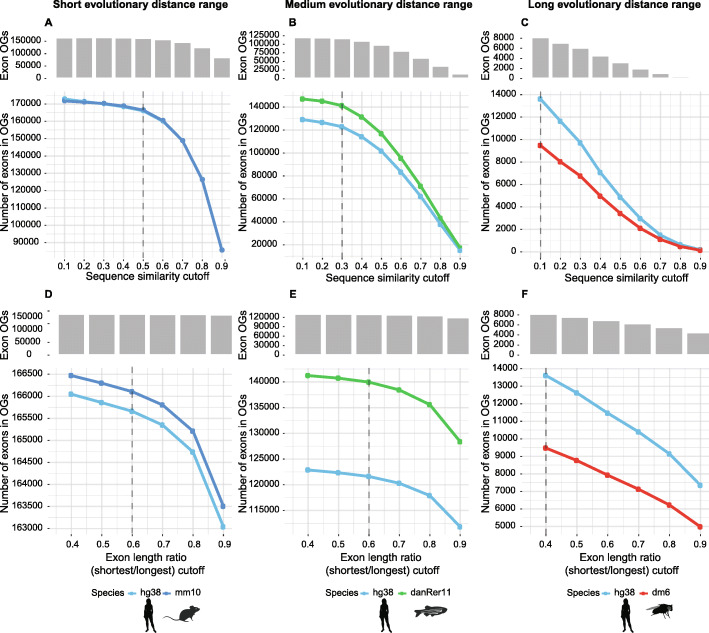
Calibration of *ExOrthist*’s conservation cut-offs. Estimation of default conservation cut-offs for each evolutionary distance range from two-species *ExOrthist* runs. The selected default conservation cut-offs are highlighted by a vertical dashed line. **a**,**d** Human and mouse (short evolutionary distance range). **b**,**e** Human and zebrafish (medium evolutionary distance range). **c**,**f** Human and fruitfly (long evolutionary distance range). **a–c** The line charts depict the number of exons from each species included in *ExOrthist* orthogroups (OGs) when different sequence similarity cut-offs are set [0.1–0.9]. The barplots on top represent the number of exon orthogroups retrieved by *ExOrthist* with the corresponding sequence similarity cut-off. All *ExOrthist* runs were performed with a fixed exon length ratio (shortest/longest) cut-off of 0.40. **d–f** The line charts depict the number of exons for each species included in *ExOrthist* orthogroups when different exon length ratio (shortest/longest) cut-offs are set [0.4–0.9]. The barplots on top represent the number of exon orthogroups retrieved by *ExOrthist* with the corresponding exon length ratio cut-off. *ExOrthist* runs were performed with the previously identified default sequence similarity cut-off (**d** 0.5, **e** 0.3, **f** 0.1). See “Methods” for details on the other settings for each run

*ExOrthist* also requires a minimum exon protein sequence similarity, or cut-off (c), for both the query exon as well as the neighboring exons, and a minimum exon length ratio, or cut-off (d), between the query exon and its match (shorter/longer). Since these two cut-offs are the main determinants of exon homology calls, we aimed at defining the most optimal combination of cut-offs for each evolutionary distance range. For this purpose, we chose human and mouse, human and zebrafish, and human and drosophila as representative species pairs for short, medium, and long evolutionary distances, respectively. We first evaluated *ExOrthist*’s performance at a fixed exon length ratio (0.40) and with a minimum exon protein sequence similarity ranging from 0.1 to 0.9 (Fig. 2A–C). Here, it is expected that lower cut-offs will retrieve higher numbers of inferred homologous relationships. Therefore, we specifically aimed at identifying the highest sequence similarity cut-off yielding nearly maximal detection sensitivity. This means the cut-off at which the vast majority of potential exon homologs would be included in the final orthogroups but for which lower cut-offs would not substantially increase the number of detected conserved exons. In the case of short and medium evolutionary distance ranges, the number of conserved exons included in orthogroups reached saturation at sequence similarity 0.5 and 0.3, respectively, with lower cut-offs leading to only minor increases in the number of exons included in the orthogroups. Thus, we selected 0.5 and 0.3 as the default minimum sequence similarity cut-offs for short and medium evolutionary distance ranges. For the human-drosophila comparison (long evolutionary distance), it was not possible to identify a saturation point, suggesting that a substantial fraction of exonic sequences has evolved beyond recognition. We therefore set the default value for minimum sequence similarity for long evolutionary distance ranges to 0.1.

Next, using these default cut-offs for the minimum sequence similarity, we evaluated the effect of varying the minimum exon length ratio from 0.4 to 0.9 for each evolutionary distance range. The goal of this analysis is thus to identify the optimal maximum relative length difference allowed between the two homologous exons for each evolutionary distance range. As above, the number of inferred exon homologs and orthogroups saturated at an intermediate value for short and medium, but not for long, evolutionary distance ranges (Fig. 2D–F). Therefore, we set the default values for minimum exon length ratio to 0.6 for short and medium and 0.4 for long evolutionary distance ranges, respectively. Data on the computing performance of *ExOrthist* for each type of run (short, medium, long), as well as for combinations of three or four species, are provided in Additional file 1: Figure S1 and in the respective *Nextflow* reports [34]. As an example, an analysis involving two vertebrate species with many annotated isoforms (e.g., human and zebrafish, with ~ 93 K and ~ 46 K protein-coding transcripts, respectively), takes ~ 1 h of run time in a CPU cluster, requiring ~ 99 h of CPU time, 4.5 GB of RAM memory, and less than 10 GB of disk space. Providing precomputed IPA alignments from previous *ExOrthist* runs (using the option *--prevaln*) reduces the necessary CPU time to less than 25 h, saving more than 75% of the required computation time. We also implemented a configuration file for running the whole analysis on the Amazon cloud. For this test case, we uploaded input data into a S3 Bucket and used an AWS Batch spot queue with a bid percentage of 50% using optimal instance type nodes. The test run took ~ 3.5 h to complete, with ~ 31 CPU hours and a total cost below 3 EUR. Thus, there is no need for having a powerful HPC for running an analysis with well-annotated vertebrate species with *ExOrthist*.

**Fig. 3:**
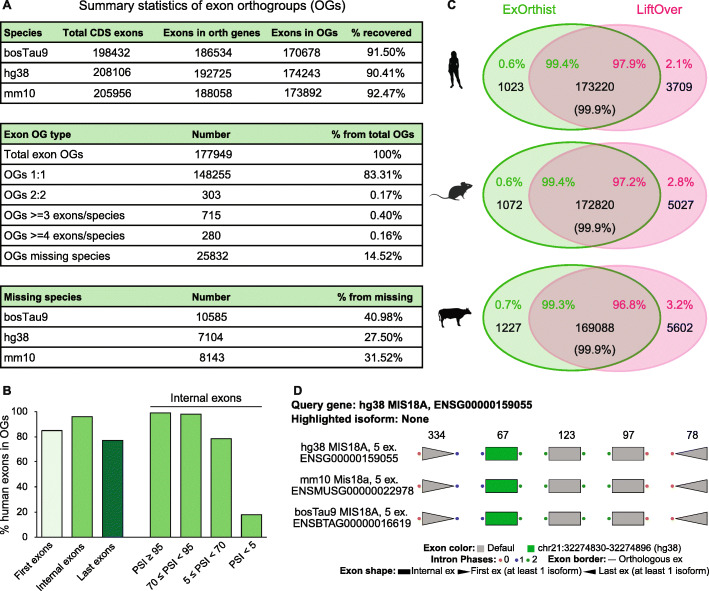
*ExOrthist* output and benchmarking. **a** Statistics of the exon orthogroups (OG) from an *ExOrthist main* run on three mammalian species (human, mouse, cow) as generated by *get_cluster_stats.pl*. **b** Percentages of conserved human coding exons based on their location within the gene (first, internal, last) or on their average inclusion level (for internal exons only), as obtained from *VastDB* [29]. **c** Venn diagrams showing the number of exons (black) included in either *ExOrthist* orthogroups (green area), *liftOver*-based orthogroups (pink area) or both (intersection) for human, mouse, and cow. The percentages of shared and not-shared exons between *ExOrthist* and *liftOver*-based orthogroups are reported in the corresponding area. The percentage in brackets refers to the concordance between the 1:1 exon pairs in the target species (e.g., mouse and cow for human exons) retrieved by both methods. **d** Plot of the exon-intron structure of human *MIS18A* and its mouse and cow orthologs as generated by the *exint_plotter* module. Exons in the different species falling in the same exon clusters are vertically aligned, and a randomly chosen exon of interest is highlighted in green. Intron positions are depicted as colored dots, depending on the phase (0: red, 1: blue, 2: green). First and last exons are shown as arrows, and the length of the CDS portion of each human *MIS18A* exon (in nucleotides) is reported on top

We also aimed at assessing the sensitivity and accuracy of *ExOrthist*’s homology calls. Although there is currently no available software to infer equivalent exon homology relationships, we compared the output of *ExOrthist* with that obtained using a *liftOver*-based approach ([35] and see “Methods”). We generated exon orthogroups for all annotated exons in human, mouse, and cow using *ExOrthist* with default cut-offs for short evolutionary distance. *ExOrthist* retrieved 148,255 1:1:1 exon triplets (83.3% of all exon orthogroups) and 25,832 (14.5%) orthogroups with a missing species (40.1% of which corresponded to cow) (Fig. 3A). Internal coding human exons showed higher levels of genomic conservation (i.e., had at least an ortholog in another species) than first and last exons (Fig. 3B), and, as expected, these conservation levels were strongly dependent on the average inclusion of the exon, with > 99% and < 18% of constitutive and cryptic internal exons having an ortholog in the other species, respectively (Fig. 3B). Importantly, the comparison with the *liftOver*-based pipeline showed a high degree of overlap (> 97%) among the exons recovered in the orthogroups by each approach (Fig. 3C), with discrepancies largely corresponding to differences in annotations of exon variants between species. Moreover, the concordance between the orthology calls was ~ 100% (i.e., the same pairs of exons from species1 and species2 were considered orthopairs by both methods) (Fig. 3C). Finally, we selected a simple gene orthogroup (*MIS18A*) to exemplify the output of the *exint_plotter* module (Fig. 3D).

### Using *ExOrthist* to investigate asymmetric exon evolution after gene duplication

**Fig. 4:**
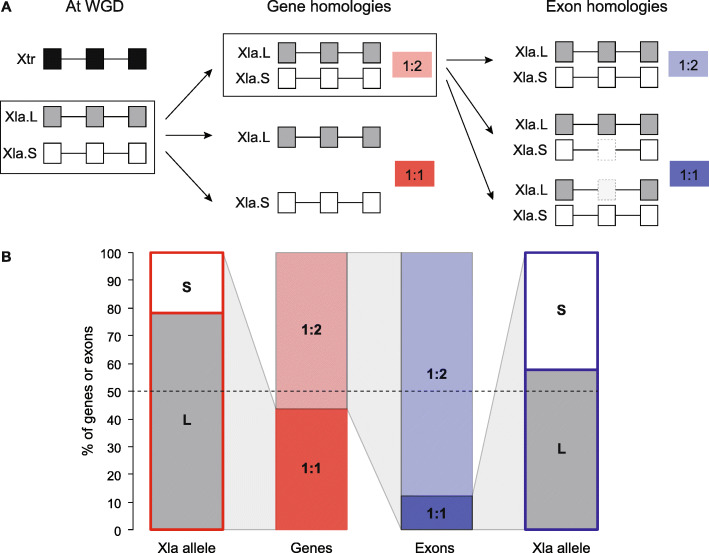
*ExOrthist* paralogy inference in the *Xenopus* genus. **a** Scheme depicting the most common fates for the *X. laevis* (Xla) paralogs (genes and exons) generated by a whole genome duplication (WGD) occurred 31 million years after the split between Xla and *X. tropicalis* (Xtr) [36]. Each copy of a paralogous gene pair belongs to a different subgenome (S or L). Both gene paralogs can be conserved, giving rise to 1:2 orthologs (Xtr:Xla). Alternatively, one gene copy can be lost (either from the S or the L subgenome), re-establishing a 1:1 orthology relationship. The same classification is applied to paralogous exons in 1:2 gene orthologs. Ancestral exons may be conserved in both genes copies (1:2) or lost by either the S or L copy (1:1). **b** Percentages of 1:1 and 1:2 genes (red) or exons (blue) in the Xla genome, with the relative percentages of S and L alleles for the 1:1 genes/exons. Cases for which the L or S membership was not defined were excluded from the plot

One important feature of *ExOrthist* is that it affords exon homology inferences not only for cases of clear 1:1 orthologs but also in scenarios with different levels of paralogy. Therefore, to assess this functionality, we performed an evolutionary exon comparison between *Xenopus tropicalis* and the allotetraploid *Xenopus laevis*. Since the whole genome duplication of *X. laevis* is relatively recent (17-18 million years ago), most of its genes can be readily assigned to one of the two ancestral subgenomes (named L and S, respectively; Fig. 4A) [36]. Although both ancestral copies (homeologs) have been retained for multiple genes, a substantial fraction has asymmetrically retained a single copy, and this has most often corresponded to the one in the L subgenome (Fig. 4B) [36]. To investigate exon evolution after whole genome duplication, we used *ExOrthist* to generate exon orthogroups within a pre-defined set of 8806 *X. leavis* homeolog pairs and their respective *X. tropicalis* single orthologs (1:2 gene orthologs) [36]. *ExOrthist* identified 69,035 (88.0%) 1:2 exon orthologs as well as 9377 (12.0%) 1:1 relationships, which indicate lack of detected exon homology for one gene homeolog in *X. laevis* after genome duplication (Fig. 4B). Interestingly, similar to the pattern of retention of gene duplicates, we found that gene copies from the L subgenome more often retained detected ancestral exon homology compared to the S subgenome (Fig. 4B; 57.7%, *P* = 7.8e−48, two-sided Binomial test).

### Evaluation of genomic and regulatory conservation of alternatively spliced exon sets

**Fig. 5:**
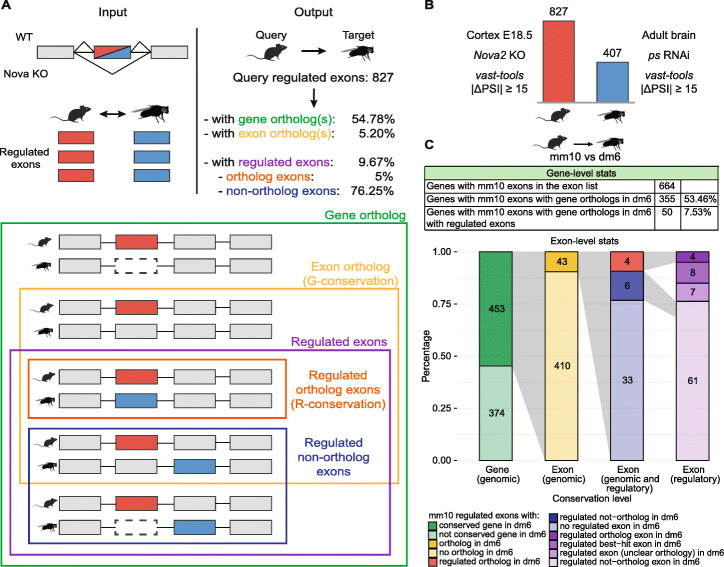
*ExOrthist compare_exon_sets* module. **a** Schematic representation of the inputs required by the *compare_exon_sets* module and of the provided output. For a given query regulated exon (red): “Gene ortholog,” the query exon belongs to a gene that has orthologs in the target species; “Exon ortholog,” the target exon has at least one valid exon ortholog as defined by *ExOrthist* in the target species (referred to as Genome conservation or G-conservation); “Regulated exons,” both the query exon and a target exon (blue) are regulated, whether they are orthologs (“Regulated exon orthologs (R-conservation)”) or not (“Regulated non-ortholog exons”). **b** Number of *Nova*-dependent exons in mouse and fruitfly as identified by *vast-tools* (*ps*: *pasilla*, drosophila homolog of mouse *Nova1* and *Nova2*). **c** Output of the *compare_exons_sets* module when run on mouse (query) and fruitfly (target) *Nova*-regulated exon sets presented in **b**. The table reports the conservation statistics at the gene level, while the barplot represents the conservation statistics at the exon level (with colors referring to the scenarios depicted in **a**)

A major application of *ExOrthist* is the assessment of the evolutionary conservation of alternatively spliced exon networks (also referred to as exon programs). These networks consist of sets of exons that are regulated in a coordinated manner in a specific tissue (e.g., brain, muscle), usually through the action of tissue-enriched alternative splicing factors, and are known to play crucial roles in cellular differentiation and physiology [37, 38]. Moreover, some sets of exons have been shown to be jointly misregulated in human pathologies, including cancer or mental disorders [39, 40], and the evolutionary comparison between human and model organisms is a powerful approach to identify potential pathogenic targets [41]. The *ExOrthist compare_exon_sets* module allows the evaluation of the evolutionary conservation of exon sets between two species from a genomic and regulatory perspective, both at the gene and exon level (Fig. 5A). At the gene level, *ExOrthist* assesses the proportion of exons in genes that have orthologs in the target species and the proportion of those orthologs that also harbor regulated exons. Similarly, exons are individually evaluated to infer what proportion have orthologs in the other species (referred to as “Genome conservation” [42]) and for what proportion those orthologs are also regulated (“Regulation conservation” [42]). Additionally, for all gene orthologs containing regulated exons in both species, the *compare_exon_sets* module performs a pairwise comparison to assess whether the exon pairs are as follows: (i) orthologs, (ii) best-hits (i.e., they are considered the best matching exon by the *main* module, but do not meet all the requirements to be considered proper homologs), (iii) non-orthologs, or (iv) unclear. In particular, *ExOrthist* relies on several aspects to confidently assign two exons as non-orthologs (Additional file 1: Figure S2), allowing the inference of evolutionarily independent alternative splicing patterns.

We used *ExOrthist* to compare sets of exons regulated by the splicing factor *Nova* in mouse and fruitfly. For this purpose, we ran *vast-tools* [29] to obtain changes in inclusion levels (ΔPSIs) for all exons upon *Nova2* depletion in mouse embryonic cortex [43] and upon knockdown of the fruifly ortholog (*ps*) in whole adult brains [44]; we then defined as *Nova*-dependent exons in each species those with a |ΔPSI| > 15 (827 in mouse and 407 in fruifly, respectively; Fig. 5B). In parallel, we ran *ExOrthist main* for mouse and fruitfly with default cut-offs for the long evolutionary distance range and providing all exons evaluated in *vast-tools* as additional exons for each species through the *--extraexons* option. We then utilized the *compare_exon_sets* module to compare the mouse and fruitfly *Nova*-dependent exon sets and identify conserved exons. Despite the expected low conservation [45, 46], *ExOrthist* identified 43 (5.2%) mouse *Nova*-dependent exons sharing orthogroups with fruitfly exons, 4 of which were also impacted by *Nova* depletion in the insect species (Additional file 2: Table S1). However, the most remarkable finding of this investigation was evidence for shared *Nova* regulation of orthologous genes through regulation of non-orthologous exons: 80 mouse exons (in 50 genes) were in gene orthologs harboring *Nova*-dependent exons in fruitfly (77 exons in 41 genes), the vast majority of which (61/80 mouse exons, 76.3%) corresponded to non-orthologous exon pairs in the two species, compared to the 4 (5%) cases with regulatory conservation and 8 (10%) additional pairs that were best exon hits although they did not pass all the homology filters (Fig. 5C and Additional file 3: Table S2). The overlap of homologous genes was much higher than expected by chance (*P* = 1.7e−13, one-sided Hypergeometric test). These results highlight a major evolutionary convergence of *Nova*-regulated exon networks impacting orthologous genes, as seen also for other splicing factor networks [46–48].

To gain further insights into the exon programs regulated by *Nova* in both lineages, we next performed an in-depth comparative analysis. First, we found that exons that were up- and down-regulated upon *Nova* disruption showed a similar neural depletion and enrichment, respectively, in both mouse and fruitfly (Additional file 1: Figure S3A,B). While *Nova*-regulated exons are well known to be neurally regulated in mammals [49], this had not been investigated in fruitflies, where *ps* expression is broader during embryo development [46, 50]. Second, we used *matt* [51] to evaluate over 50 splicing-related features in both species. Compared to non-regulated alternative exons and other exon sets in their corresponding species, *Nova*-regulated exons had overall similar features, including shorter exon lengths (Additional file 1: Figure S3C-G). A notable exception was the length of poly-pyrimidine tract of the upstream intron, which was longer in mouse but shorter than for other exon sets in fruitflies (Additional file 1: Figure S3H). Finally, we also used *matt* to identify enriched motifs from RNA-binding proteins potentially associated with *Nova* regulation. As expected, *Nova*-regulated exons from both species exhibited strong enrichment for canonical YCAY clusters in the expected positions based on the direction of regulation (Additional file 1: Figure S4A,B) [45, 52]. Interestingly, we also found enrichment for Rbfox-binding motifs downstream of mouse *Nova*-dependent exons (Additional file 1: Figure S4C), in line with previous reports [53]. However, this enrichment was not observed for flies, suggesting the co-regulation by both factors is lineage-specific (Additional file 1: Figure S4D). Conversely, *Nova*-regulated exons in *Drosophila*, but not in mouse, exhibited enrichment for motifs for *IGF2BP2/Imp* (Additional file 1: Figure S4E,F), an RNA-binding protein upregulated during early neuronal differentiation in both lineages (Additional file 1: Figure S4G,H).

## Discussion

Exon gains and losses have the potential to drive diversification of gene structure and function across eukaryotes. Moreover, alternative splicing of specific exons can greatly expand transcriptome and proteome diversity, especially in metazoans. In this context, evolutionary conservation both at the genomic and the regulatory level of alternative splicing has been widely regarded as a proxy for functional relevance [42]. A key step to assess such conservation is the inference of exon homology relationships among species. However, while multiple tools have been developed for gene homology inference, there is currently no available software to derive exon orthology and paralogy relationships. Here, we have introduced *ExOrthist*, the first software specifically designed to infer exon homology relationships taking into account the unique characteristics of exon and intron evolution across different evolutionary distances. Therefore, unlike gene-oriented software, *ExOrthist* does not rely on comparisons of exon sequence similarity in isolation, but also makes use of the full intragenic context. For this reason, *ExOrthist* identifies potential exon homologs through global protein alignments and explicitly requires conservation of the neighboring intron positions as well as of individual exon sequences within these global alignments.

Evolution of intron positions has been extensively studied, revealing a generally low rate of intron gain and loss within most eukaryotic groups [54–57], despite some remarkable exceptions (e.g., [58, 59]). Comparison among lineages showed conservation of relatively high fractions of ancestral introns in various clades, as well as substantial remodeling of intron-exon architectures [56, 60]. Since, by definition, intron insertions define (and redefine) exons, conservation of ancestral intron positions is a requirement for true exon homology of any given exon pair. Thus, because of their very low rates of intron gains/losses, it is expected that a large fraction of exons is conserved within vertebrates (Fig. 3, [5, 61, 62]), but not between species with largely divergent intron-exon architectures (e.g., between mammals and dipterans; Fig. 5). That is, even if the compared coding sequences are orthologous, the actual exon entities encoding them may not be. To our knowledge, *ExOrthist* is the first framework that incorporates the conservation of intron positions and other features of the gene context to explicitly draw the fundamental distinction between conserved exon sequences and conserved exon entities. In order to clearly distinguish between these two scenarios, *ExOrthist* also provides the best hit for each exon within each target gene homolog, even when the target exon does not meet the necessary requirements to be considered a homolog. As we have shown in the case of *Nova*-regulated exons, this allows the identification of cases of convergent evolution of (regulated) alternative splicing, in which two exons that are not orthologous, even if sometimes they partially share orthologous sequences, are independently recruited to corresponding exon networks in two lineages.

Since *ExOrthist* allows users to work at any evolutionary distance and with species for which only a genome sequence and a standard gene annotation is available, our software can be used for both model and non-model organisms. For this reason, we envision that *ExOrthist* will enable unprecedented evolutionary studies, both in terms of species representation and biological questions, opening multiple new venues of research on genome evolution and alternative splicing. To name a few, these could include the identification of deeply conserved alternative splicing events, the study of the origin and evolution of tissue-specific exon networks, the investigation of intragenic evolutionary patterns, the inference of phylogenies based on *bona fide* exon orthologs, and the construction of large multi-species databases interconnected not only through gene homology relationships (e.g., *Ensembl*), but also through exon homology relationships (e.g., *VastDB* [29]).

## Conclusions

*ExOrthist* is the first tool specifically designed to infer exon orthogroups and orthopairs across any evolutionary distance. It also contains additional modules to visualize conservation of intron-exon architectures and to assess evolutionary conservation of alternative splicing patterns. *ExOrthist* will thus facilitate research on genome evolution at the intragenic level as well as the identification of highly conserved, and thus more likely functional, alternatively spliced exons.

## Methods

### Algorithm of *ExOrthist main* module

The *ExOrthist main* module requires a minimum set of inputs (annotation files, genome files, evolutionary distance information, and gene orthogroups) to return the complete set of exon orthopairs and orthogroups for a chosen group of species. The following sections (A, B, C, D) describe the algorithm applied by *ExOrthist*, as well as the extra steps implemented when additional inputs are provided. The module relies on software and libraries stored within a docker image whose recipe is bundled together with the rest of the pipeline. The image is available at docker hub (https://hub.docker.com/r/biocorecrg/exon_intron_pipe) and is automatically retrieved by *ExOrthist* and eventually converted into a singularity image if the option *-with-singularity* is specified. To run the program, a user only needs to install *Nextflow* and one Linux container, either *Docker* or *Singularity*. Further details can be obtained in the README section at https://github.com/biocorecrg/ExOrthist.

A. Input generation

After checking for the presence of all required inputs and the consistency between gene orthogroups and the annotated gene IDs in the GTF files, *ExOrthist* generates files with annotation information for each considered species, which will be the input of all species pairwise comparisons (see online README for a detailed list of all the files). Although, by default, only annotated exons are considered, *ExOrthist* can integrate non-annotated exons into its homology inferences by providing a list of gene identifiers plus exon coordinates (e.g., exons identified by *vast-tools* [29] or any other software to detect and/or quantify alternative splicing). If non-annotated exons are provided, *ExOrthist* generates a new GTF file where a novel transcript for each of the non-annotated exons is introduced. *ExOrthist* first maps the upstream and downstream exons (provided in the input file) to the annotated transcripts. When both neighboring exons match the same coding transcript, this becomes the template of the novel transcript to which the non-annotated exon is added; otherwise, *ExOrthist* uses partial matches of the upstream or downstream exon to identify the template transcript and create the new annotation.

*ExOrthist* allows the user to specify different conservation cut-offs depending on the evolutionary distances between the compared species, which have to be declared in a specific input file. In particular, four evolutionary cut-offs can be fine-tuned for three evolutionary distance ranges (short, medium, long): (a) minimum global protein sequence similarity for a pairwise isoform alignment to be considered for the orthology call, (b) minimum number of conserved intron positions, (c) minimum sequence similarity required for orthologous exons, and (d) minimum ratio between the lengths of an exon pair (length of the shortest/longest exon). Cut-offs (b), (c), and (d) will be utilized in the later steps of the pipeline to filter out non-homologous matches from the pool of candidate exon orthopairs for each pair of species (see section C).

B. Parsing of IPA alignments

*ExOrthist* then works by species pairs and within gene orthogroups. For each species pair and gene orthogroup, it first generates pairwise Intron Position Aware (IPA) alignments between all non-redundant protein isoforms of species1 vs. all non-redundant protein isoforms of species2 (Fig. 1C). Protein sequence alignments are generated using *MAFFT* v7 [63] and intron positions are introduced subsequently. Considering species1 as query and species2 as target (and vice versa), *ExOrthist* parses the IPA alignments to derive:

- Protein conservation information: species-wise percentages of sequence identity, sequence similarity (based on BLOSUM62), and gaps between the two proteins are calculated. Alignments are not considered for further processing unless the global sequence similarity (cut-off (a)) reaches the minimum protein similarity specified for the relative evolutionary distance (see section A) for both species, or twice the minimum protein similarity cut-off (2 × cut-off (a)) for one of the species.
- Exon conservation information: for each query exon, *ExOrthist* selects all exon matches in all target isoforms. In case of a query exon matching multiple target exons in the same isoform each covering ≥ 15% of the query exon sequence, each exon pair is specifically realigned and the best match is selected based on sequence similarity.
- Intron conservation information: *ExOrthist* tries to match each query intron with a target intron in the IPA alignment. Depending on the local protein similarity, the width of the searching window around the query intron changes:
  - if % similarity ≤ 30 or % gaps ≥ 30: width = 10
  - if % similarity ≥ 30 and % similarity < 50: width = 8
  - if % similarity ≥ 50 and % similarity < 70: width = 6
  - if % similarity ≥ 70 and % similarity < 80: width = 4
  - if % similarity ≥ 80 and % similarity < 90: width = 3
  - if % similarity ≥ 90: width = 2
  This window is further increased by the average number of gaps (i.e., “-”) within ten positions at each side of the alignment. Then, if a target intron is not found within the search window, conservation of the query intron is set to zero. If a match is found and the phases of the two introns are equal, intron conservation is rated 10. If the phases of the two introns are different, intron conservation is rated − 10. Next, if the two intron positions are not perfectly aligned within the protein sequence alignment, the number of residues by which they are separated in the alignment is subtracted to the intron conservation value. Thus, the maximum intron conservation value is equal to 10 for a perfectly aligned pair of introns in the same phase, and decreases progressively to indicate no intron conservation (0) or presence of non-conserved introns with different phase (negative values). The intron position conservation value is later translated into a partial score used to evaluate pairwise exon orthology relationships. Importantly, intron position conservation is solely evaluated based on the position the introns occupy in the protein alignment and their phases. Other features such as intronic sequence or length are not taken into account, since they are often poorly conserved.

*ExOrthist* efficiently parallelizes all alignments and realignments thanks to *Nextflow*’s management system, and later joins all the best isoform-wise matches for a given species pair. If the output of a previous *ExOrthist* run is provided (*--prevaln* option), the alignment and realignment steps are skipped for all the query and target isoform pairs that have already been aligned.

C. Scoring and best match selection

*ExOrthist* keeps executing the processes separately for each species pair. In here, it selects the best match per gene for each exon in the query species among all the isoform matches of each homologous gene in the target species. Priority is given to the target exons that fulfill the required homology criteria (see below), which are evaluated by five partial scores reflecting the conservation of the previously assessed features of the exon-intron context (section B):

- (1)–(2): Conservation of the (1) upstream and (2) downstream intron phases: ranging [−0.25, 0.25], obtained from multiplying the intron conservation value from B by 0.025. Positive values indicate phase conservation, while deviations from the maximum score reflect shifts in the intron positions within the IPA.
- (3): Conservation of the exon sequence: ranging [0, 0.2]. 0.2 indicates 100% sequence similarity.
- (4)–(5): Conservation of the (4) upstream and (5) downstream exon sequences: ranging from [0, 0.15]. 0.15 indicates 100% sequence similarity.

These partial scores for internal exons are then summed up to generate a global score ranging [0,1]. In the case of first or last exons, for which scores (2) and (5) or (1) and (4) do not exist, respectively, the global score is divided by 0.6 to make it range [0,1]. Importantly, the global score is used to select the best representative target isoform per query exon, but is not directly used to infer homology. For the homology inference, different filtering criteria will be applied to each species pair based on their evolutionary distance and the user-specified conservation cut-offs for each of the individual scores. More specifically, the up/downstream intron position conservation, the exon sequence conservation and the up/downstream exon sequence conservation are sequentially evaluated in the filtering.

- (1) and/or (2), depending on cut-off (b), are required to be positive in all the target-gene best matches (i.e., the intron positions need to be in the same phase and within the allowed window, even if not necessarily in the exact same location). For first and last exons, only (2) and (1) are evaluated, respectively. If the intron phase(s) are conserved, sequence conservation is evaluated.
- (3) is required to be ≥ min_ex_sim*0.2, where *min_ex_sim* is the minimum sequence similarity specified for species1-species2 evolutionary distance range (cut-off (c)).
- (4) and (5) are required to be ≥ min_ex_sim*0.15, where *min_ex_sim* is the minimum sequence similarity specified for species1-species2 evolutionary distance range (cut-off (c)). For first and last exons, only (5) and (4) are evaluated, respectively.
- Extra filter: the length ratio between two compared exons (shortest/longest) should not be lower than the minimum exon length ratio specified for species1-species2 evolutionary distance range (cut-off (d)).

In the end, among these pre-filtered matches, the match with the highest global score is selected for each query exon and target gene. If no target exon passes all cut-offs, the one with the highest global score is considered the best hit for the target gene. While the *ExOrthist*’s logic requires a query exon to match a unique target exon, each target exon can potentially be matched by multiple query exons, capturing cases of in-tandem exon duplication in the same gene. Moreover, overlapping variants of the query exons (i.e., alternative forms of the same exon due to alternative splice donor/acceptor sites) might be present in the pool of filtered matches. In order to univocally derive orthologous relationships for each exon, *ExOrthist* selects the form with the highest number of matches (among all target genes) as representative of its overlap group. The best pairwise IPA alignment for a pair of exons can be automatically retrieved using the script *retrieve_IPA_aln.pl*.

In addition, *ExOrthist* accepts custom exon orthopairs (from *liftOver* or manual curation) to be considered in the final exon orthology inference through the *--bonafide_pairs* option. For this purpose, *ExOrthist* incorporates a custom script (*get_liftovers.pl*) to allow users to retrieve *liftOver*-based exon orthopairs for species pairs with available *liftOver* files. If provided, these exon orthopairs will be given priority over the *ExOrthist*-inferred matches (i.e., they will be chosen as representatives of their overlap groups) and will be directly integrated in the generation of the exon orthogroups graphs (next section).

D. Exon clustering

After deriving exon homologous relationships between each pair of species, *ExOrthist* works on the combined orthopair information to infer the exon orthogroups. For each gene orthogroup, *ExOrthist* builds a directed graph (through the R *igraph* package v1.2.6 [32]) with exons as nodes and their pairwise homology relationships represented as edges. In case of a best reciprocal match between homologous exons, two directed edges will be drawn between the correspondent nodes. *ExOrthist* then applies the R *igraph* edge-betweenness algorithm to select the optimal graph topology, with communities highly intra-connected and lowly interconnected. Although the directionality of the graph is not considered by the edge-betweenness algorithm, the reciprocity of the matches is represented by the number of edges. The exon communities identified in the optimal topology correspond to the multi-species exon orthogroups returned by the pipeline. For each exon, *ExOrthist* also computes a Membership Score (MS), which reflects its degree of similarity to all the exons belonging to the same orthogroup (OG). The MS is defined as follows:

MS = (IN_degree + OUT_degree + N_reciprocals) / (2*(TOT_exons_in_OG - SPECIES_exons_in_OG) + (TOT_genes_in_OG - SPECIES_genes_in_exOG))

with:

- IN_degree: number of exon matches from the other exons in the orthogroup (i.e., the considered exon is target).
- OUT_degree: number of exon matches to the other exons in the orthogroup (i.e., the considered exon is query).
- N_reciprocal: number of reciprocal matches (i.e., query exon is a match of its target exon and vice versa).
- TOT_exons_in_OG: number of exons in the orthogroup.
- SPECIES_exons_in_OG: number of exons from the same species present in the exon orthogroup.
- TOT_genes_in_OG: total number of genes in the original gene orthogroup.
- SPECIES_genes_in_exOG: number of genes from the same species that contribute with exons to the exon orthogroup.

### Genome annotation and sequence files

In order to run *ExOrthist*, annotation files (GTF) and genome files (fasta) have to be provided for each of the species of interest. For the analyses presented in this study, we downloaded the GTF and genome fasta files from Ensembl (https://www.ensembl.org/), selecting the following assemblies and versions: human (hg38, v88), mouse (mm10, v88), cow (bosTau9, v99), and zebrafish (danRer11, v99). For fruitfly, we downloaded the GTF and fasta genome files from Ensembl Metazoa (dm6, v26). To comply with the UCSC Brower’s format, we modified the first field of all GTF files so that the label “chr” was added to the chromosome number (e.g., “1” = > “chr1”).

### Calibration of *ExOrthist*’s default conservation cut-offs

We first selected three pairs of species as representatives of each evolutionary distance range. Human-mouse for short (hg38-mm10; estimated divergence: 90 million years ago [MYA] [64]), human-zebrafish for medium (hg38-danRer11; estimated divergence: 435 MYA [64]), and human-fruitfly for long (hg38-dm6; estimated divergence: 797 MYA [64]). We then used *Broccoli* v1.0 with default settings [18] to infer each pairwise gene orthogroups. We removed all the gene orthogroups containing more than 20 genes, selecting 16,034 orthogroups for hg38-mm10, 11,571 for hg38-danRer11 and 5142 for hg38-dm6.

In order to identify the optimal minimum exon sequence similarity (cut-off or parameter (c)) and minimum exon length ratio (shortest/longest) (cut-off or parameter (d)), we systematically analyzed the *ExOrthist main* (v0.1.0) performance with different combinations of those parameters. First, we kept the minimum length ratio fixed at 0.4 and we performed nine *ExOrthist* runs for each species pair starting from a minimum sequence similarity of 0.1 and increasing it by 0.1 in each of the following runs up to 0.9. We then selected the optimal sequence similarity cut-off for the short and medium evolutionary distance ranges depending on when saturation in the number of conserved exons was reached (0.5 and 0.3, respectively) (Fig. 2A–C). For long evolutionary distances, we selected 0.1 as default sequence similarity cut-off, since saturation was not reached.

Second, we kept the minimum sequence similarity fixed at the identified default parameter for each evolutionary distance range, and we performed five *ExOrthist* runs for each species pair starting from a minimum exon length ratio of 0.4 and increasing it by 0.1 in each of the following runs up to 0.9. The saturation in the number of conserved exons was reached at an exon length ratio cut-off of 0.6 for both short and medium evolutionary distance ranges. For long evolutionary distances, we selected 0.4 as the default exon length ratio cut-off, since saturation was not reached (Fig. 2D–F). All the mentioned runs were executed with minimum protein similarity = 0.25 (short), 0.20 (medium), 0.15 (long), and fixed minimum number of conserved introns = 2

### Evaluation of *ExOrthist* computational performance

To assess *ExOrthist* computational performance, we ran the *main* module (v1.0.0) for the species pairs representative of each evolutionary distance [short: human-mouse (hg38-mm10), medium: human-zebrafish (hg38-danRer11), long: human-fruitfly (hg38-dm6)], setting the identified optimal conservation cut-offs (minimum sequence similarity and minimum length ratio; see previous paragraph), fixed number of introns = 2 and protein similarity = 0.25 (short), 0.20 (medium), and 0.15 (long). Moreover, to evaluate how the required computational hours and running time scale when launching *ExOrthist* on more than two species, we performed two extra runs considering three (human: hg38, mouse: mm10, zebrafish: danRer11) and four (human: hg38, mouse: mm10, zebrafish: danRer11, fruitfly: dm6) species at a time. We first used *Broccoli* v1.0 with default settings [18] to infer gene orthogroups in each set of species. We removed all the gene orthogroups containing more than 20 genes, selecting 15,712 orthogroups for hg38-mm10-danRer11 and 12,509 orthogroups for hg38-mm10-danRer11-dm6. We then ran *ExOrthist main* (v1.0.0) with the conservation parameters specified above, and by setting the following species pairwise evolutionary distances: short (hg38-mm10), medium (hg38-danRer11, mm10-danRer11), long (hg38-dm6, mm10-dm6, danRer11-dm6). Lastly, we repeated the runs by providing precomputed pairwise protein alignments through the *–prevaln* option. Comparisons of the computational performance of these multi-species runs, together with that of species pairwise runs, are reported in Additional file 1: Figure S1.

### Comparison of *ExOrthist* and *liftOver* performances

To benchmark *ExOrthist* at a short evolutionary distance range, we implemented a *liftOver*-based approach following previous studies [29, 35]. Briefly, we downloaded the *liftOver* chain alignment files for each pairwise comparison (among human, mouse and cow; hg38, mm10 and bosTau9) and performed the *liftOver* for all annotated exons from each species against the other two. As recommended by the developers, we used the following parameters: -minMatch = 0.10 -multiple -minChainT = 200 -minChainQ = 200, which we implemented in the script *get_liftovers.pl* provided as part of *ExOrthist*. Then, to make the comparisons between *ExOrthist* and *liftOver* fairer, we did not require the *liftOver* matches to have any canonical dinucleotide, and we considered only lifted coordinates that shared a junction with an annotated exon in the target species and in the same gene orthogroups used to run *ExOrthist*. From these exon pairs, we generated exon orthogroups by running the final scripts of the *ExOrthist main* module (v0.1.0): *D2_cluster_EXs.R* and *D3_format_EX_clusters_output.pl*.

To obtain *ExOrthist* exon orthogroups, we used *Broccoli* v1.0 [18] with default settings to infer gene orthogroups for human, mouse and cow. We filter out 53 orthogroups containing more than 20 genes, restricting the following analysis to 17,594 orthogroups. We ran *ExOrthist main* (v0.1.0) with conservation cut-offs set at the default values for short distance species (minimum sequence similarity = 0.5; minimum exon length ratio = 0.6, minimum global protein similarity = 0.25, and minimum number of conserved introns = 2).

To compare the exon orthogroups from both approaches, we performed two analyses. First, we asked, for each species and for each approach, how many annotated exons were found in orthogroups with exons from each of the other two species and plotted the overlapping fraction (Fig. 3C). Since exons may have alternative splice acceptor and/or donor sites, we used *bedtools intersect* to compute the overlap between the two orthogroup files. Second, for exons with a 1:1 match in another species in both exon orthogroup files, we assessed the concordance of these orthology assignments (i.e., whether an exon in species1 was paired to the same exon in species2 by both approaches), obtaining virtually 100% concordance.

### Investigation of exon evolution after whole genome duplication

We obtained the 1:2 and 1:1 gene orthogroups between *X. tropicalis* (v9.0) and *X. laevis* (v9.1) from [36] and the respective genome annotations from Xenbase. Genome annotations were downloaded as GFF3 files and converted to GTF format using *gffread* [65] and custom scripts. We generated exon orthogroups by running *ExOrthist main* (v0.1.0) with default conservation cut-offs for short evolutionary distance (minimum global protein similarity = 0.25, number of required introns = 2, minimum sequence similarity = 0.5; minimum length ratio = 0.6), and we subsequently quantified the exon orthogroups (1:2 and 1:1) generated from the 8806 1:2 gene orthogroups. More specifically, 1:2 exon orthogroups contained one exon for *X. tropicalis* and its two orthologs in *X. laevis* (which derived from a whole genome duplication event). Conversely, in 1:1 exon orthogroups, only one *X. laevis* ortholog was detected. Information about the *X. laevis* subgenome (L or S) was obtained from [36] and was used to parse the 1:1 gene and exon orthogroups to assess in which subgenome the gene/exon ortholog was not detected. We considered an exon to have 1:1 or 1:2 homologs only based on the exon orthogroups and did not take the best hit information into account.

### Comparison of *Nova*-dependent exon sets

We used *Broccoli* v1.0 [18] with default parameters to infer mouse-fruitfly (mm10-dm6) gene orthogroups. We selected all the orthogroups (5207) containing less than 20 genes. We ran *ExOrthist main* (v0.1.0) with default conservation cut-offs for long distance evolutionary range (minimum global protein similarity = 0.15, number of required introns = 2, minimum sequence similarity = 0.1; minimum length ratio = 0.4), adding non-annotated exons identified by *vast-tools* [29]. To identify *Nova*-dependent exons, we run *vast-tools* v2.5.1 for two datasets characterized by deficiency of Nova orthologs in each species. In particular, we used an experiment with *Nova2* depletion in mouse embryonic cortex at the E18.5 stage [43] and another one with downregulation of *pasilla* (*ps*) in adult fly whole brains [44]. To obtain inclusion levels (PSIs) for all exons, we employed *vast-tools align* and *combine* with default parameters for mm10 and dm6 (VASTDB libraries: vastdb.mm2.23.06.20.tar.gz and vastdb.dme.23.06.20.tar.gz) [29]. All replicates for control and depleted samples were merged into a single sample using *vast-tools merge* to increase read depth, and *vast-tools compare* with default parameters (minimum change in inclusion levels [min_dPSI] of 15) was employed to define *Nova*-dependent exons (827 exons in mouse and 407 in fruitfly, respectively; Fig. 5B). Evolutionary comparison of both sets was done using the *ExOrthist compare_exon_sets* (v1.0.0) module with default options and providing two exon sets. The overlap at the gene orthology level between the sets of *Nova*-regulated exons in the two species was assessed using a hypergeometric test (*phyper* in R), considering only exons in genes included in the gene orthogroups. The following arguments were inferred: *q*, number of orthogroups with *Nova*-regulated exons in both species (41); *m*: number of orthogroups with *Nova*-regulated exons in mouse (320); *n*: number of orthogroups without *Nova*-regulated exons in mouse (5104); *k*: number of orthogroups with *Nova*-regulated exons in fruifly (193).

Neural regulation (difference in average PSI of neural and non-neural samples) for each *Nova*-regulated exon was calculated using data from *VastDB* for mm10 and dm6 [29], and through the script *Get_Tissue_Specific_AS.pl* [66]. Analysis of splicing-related features was performed with *matt cmpr_exons* [51] v1.3.0 and a selection of features was plotted in Additional file 1: Figure S3. All the represented features were directly quantified by *matt cmpr_exons* with the exception of the average intron length, which we obtained by averaging the length of the upstream and downstream introns returned by *matt*. Control exon sets for each species (constitutive, cryptic, and alternative exons not regulated by *Nova*) were obtained by filtering the output of *vast-tools compare* (run with the --print_sets option) to up to 1000 random exons per category. Finally, enrichment of RNA-binding protein motifs was done using *matt rna_maps* v1.3.0 with the REGEXP motif option for Nova ([CT]CA[CT](.{0,3}[CT]{0,1}CA[CT]|.{4,23}[CT]CA[CT]){2}, obtained from [51]), Rbfox (TGCATG), and IGF2BP2 ([A|C|G][A|C]A[A|C|T][A|T]CA, built by *matt rna_maps_cisbp* from the CISBP-RNA motif M032 [67]).

## Supplementary Information

**Additional file 1.** Contains Figures S1-S4.

**Additional file 2. **Contains Table S1 (Orthogroups output from *compare_exon_sets*).

**Additional file 3: Table S2.** (Pairwise comparison output from *compare_exon_sets*).

**Additional file 4.** Review history.

## Data Availability

The *ExOrthist* is an open software publicly available under a MIT license on Github (https://github.com/biocorecrg/ExOrthist) [34] and Zenodo [68]. For the analyses presented in this study, the GTF and genome fasta files from Ensembl (https://www.ensembl.org/) were downloaded, the following assemblies and versions: human (hg38, v88), mouse (mm10, v88), cow (bosTau9, v99) and zebrafish (danRer11, v99) were selected. For fruitfly, the GTF and fasta genome files from Ensembl Metazoa (dm6, v26) were downloaded.

## References

[1] Sanz L, Calvete JJ (2016). Insights into the evolution of a snake venom multi-gene family from the genomic organization of Echis ocellatus SVMP genes. Toxins.

[2] Cosby RL, Judd J, Zhang R, Zhong A, Garry N, Pritham EJ, Feschotte C (2021). Recurrent evolution of vertebrate transcription factors by transposase capture. Science.

[3] Grau-Bove X, Ruiz-Trillo I, Irimia M (2018). Origin of exon skipping-rich transcriptomes in animals driven by evolution of gene architecture. Genome Biol.

[4] Reyes A, Anders S, Weatheritt RJ, Gibson TJ, Steinmetz LM, Huber W (2013). Drift and conservation of differential exon usage across tissues in primate species. Proc Natl Acad Sci U S A.

[5] Barbosa-Morais NL, Irimia M, Pan Q, Xiong HY, Gueroussov S, Lee LJ, Slobodeniuc V, Kutter C, Watt S, Colak R, Kim T, Misquitta-Ali CM, Wilson MD, Kim PM, Odom DT, Frey BJ, Blencowe BJ (2012). The evolutionary landscape of alternative splicing in vertebrate species. Science.

[6] Merkin J, Russell CB, Chen P, Burge CB (2012). Evolutionary dynamics of gene and isoform regulation in Mammalian tissues. Science.

[7] Torres-Méndez A, Bonnal S, Marquez Y, Roth J, Iglesias M, Permanyer J, Almudí I, O’Hanlon D, Guitart T, Soller M, Gingras AC, Gebauer F, Rentzsch F, Blencowe BJ, Valcárcel J, Irimia M (2019). A novel protein domain in an ancestral splicing factor drove the evolution of neural microexons. Nature Ecol Evol.

[8] Gracheva EO, Cordero-Morales JF, González-Carcacía JA, Ingolia NT, Manno C, Aranguren CI, Weissman JS, Julius D (2011). Ganglion-specific splicing of TRPV1 underlies infrared sensation in vampire bats. Nature.

[9] Gueroussov S, Gonatopoulos-Pournatzis T, Irimia M, Raj B, Lin ZY, Gingras AC, Blencowe BJ (2015). An alternative splicing event amplifies evolutionary differences between vertebrates. Science.

[10] Tress ML, Abascal F, Valencia A. Alternative splicing may not be the key to proteome complexity. Trends Biochem Sci. 2017. 10.1016/j.tibs.2016.08.008 PMC6526280 27712956

[11] Blencowe BJ (2017). The relationship between alternative splicing and proteomic complexity. Trends Biochem Sci.

[12] Marlétaz F, Firbas PN, Maeso I, Tena JJ, Bogdanovic O, Perry M, Wyatt CD, de la Calle-Mustienes E, Bertrand S, Burguera D (2018). Amphioxus functional genomics and the origins of vertebrate gene regulation. Nature.

[13] Gabaldón T, Koonin EV (2013). Functional and evolutionary implications of gene orthology. Nat Rev Genet.

[14] Train C, Glover NM, Gonnet GH, Altenhoff AM, Dessimoz C (2017). Orthologous Matrix (OMA) algorithm 2.0: more robust to asymmetric evolutionary rates and more scalable hierarchical orthologous group inference. Bioinformatics.

[15] Li L, Stoeckert CJJ, Roos DS (2003). OrthoMCL: identification of ortholog groups for eukaryotic genomes. Genome Res.

[16] Miller JB, Pickett BD, Ridge PG (2019). JustOrthologs: a fast, accurate and user-friendly ortholog identification algorithm. Bioinformatics.

[17] Emms DM, Kelly S (2019). OrthoFinder: phylogenetic orthology inference for comparative genomics. Genome Biol.

[18] Derelle R, Philippe H, Colbourne JK. Broccoli: combining phylogenetic and network analyses for orthology assignment. Mol Biol Evol. 2020;msaa159. 10.1093/molbev/msaa159 32602888

[19] Zea DJ, Laskina S, Baudin A, Richard H, Laine E. Assessing conservation of alternative splicing with evolutionary splicing graphs. bioRxiv. 2020. 10.1101/2020.1111.1114.382820. 10.1101/gr.274696.120 PMC8327911 34266979

[20] Chakraborty A, Ay F, Davuluri RV. Exon- and Transcript-level mappings for orthologous gene pairs. Bioinformatics. 2021;btab393. 10.1093/bioinformatics/btab393 PMC8545320 34014317

[21] Pavesi G, Zambelli F, Caggese C, Pesole G (2008). Exalign: a new method for comparative analysis of exon-intron gene structures. Nucleic Acids Res.

[22] De Moerlooze L, Spencer-Dene B, Revest JM, Hajihosseini M, Rosewell I, Dickson C (2000). An important role for the IIIb isoform of fibroblast growth factor receptor 2 (FGFR2) in mesenchymal-epithelial signalling during mouse organogenesis. Development.

[23] Hatje K, Rahman R, Vidal RO, Simm D, Hammesfahr B, Bansal V, Rajput A, Mickael ME, Sun T, Bonn S, Kollmar M (2017). The landscape of human mutually exclusive splicing. Mol Syst Biol.

[24] Irimia M, Maeso I, Gunning PW, Garcia-Fernandez J, Roy SW (2010). Internal and external paralogy in the evolution of Tropomyosin genes in metazoans. Mol Biol Evol.

[25] Hinrichs AS, Karolchik D, Baertsch R, Barber GP, Bejerano G, Clawson H, Diekhans M, Furey TS, Harte RA, Hsu F, Hillman-Jackson J, Kuhn RM, Pedersen JS, Pohl A, Raney BJ, Rosenbloom KR, Siepel A, Smith KE, Sugnet CW, Sultan-Qurraie A, Thomas DJ, Trumbower H, Weber RJ, Weirauch M, Zweig AS, Haussler D, Kent WJ (2006). The UCSC Genome Browser Database: update 2006. Nucleic Acids Res.

[26] Tommaso PD, Chatzou M, Floden EW, Barja PP, Palumbo E, Notredame C (2017). Nextflow enables reproducible computational workflows. Nat Biotechnol.

[27] Trapnell C, Roberts A, Goff L, Pertea G, Kim D, Kelley DR, Pimentel H, Salzberg SL, Rinn JL, Pachter L (2012). Differential gene and transcript expression analysis of RNA-seq experiments with TopHat and Cufflinks. Nat Protoc.

[28] Vaquero-Garcia J, Barrera A, Gazzara MR, Gonzalez-Vallinas J, Lahens NF, Hogenesch JB, Lynch KW, Barash Y (2016). A new view of transcriptome complexity and regulation through the lens of local splicing variations. Elife.

[29] Tapial J, Ha KCH, Sterne-Weiler T, Gohr A, Braunschweig U, Hermoso-Pulido A, Quesnel-Vallières M, Permanyer J, Sodaei R, Marquez Y, Cozzuto L, Wang X, Gómez-Velázquez M, Rayon T, Manzanares M, Ponomarenko J, Blencowe BJ, Irimia M (2017). An atlas of alternative splicing profiles and functional associations reveals new regulatory programs and genes that simultaneously express multiple major isoforms. Genome Res.

[30] Shen S, Park JW, Lu ZX, Lin L, Henry MD, Wu YN, Zhou Q, Xing Y (2014). rMATS: robust and flexible detection of differential alternative splicing from replicate RNA-Seq data. Proc Natl Acad Sci U S A.

[31] Sterne-Weiler T, Weatheritt RJ, Best AJ, Ha KCH, Blencowe BJ (2018). Efficient and accurate quantitative profiling of alternative splicing patterns of any complexity on a laptop. Mol Cell.

[32] Csardi G, Nepusz T. The igraph software package for complex network research: InterJournal, Complex Systems; 2006.

[33] Irimia M, Roy SW (2008). Spliceosomal introns as tools for genomic and evolutionary analysis. Nucleic Acids Res.

[34] Marquez Y, Mantica F, Cozzuto L, Burguera D, Hermoso-Pulido A, Ponomarenko J, et al. ExOrthist: a tool to infer exon orthologies at any evolutionary distance. Github. 2021; https://github.com/biocorecrg/ExOrthist. 10.1186/s13059-021-02441-9 PMC8379844 34416914

[35] Irimia M, Weatheritt RJ, Ellis J, Parikshak NN, Gonatopoulos-Pournatzis T, Babor M, Quesnel-Vallières M, Tapial J, Raj B, O’Hanlon D (2014). A highly conserved program of neuronal microexons is misregulated in autistic brains. Cell.

[36] Session AM, Uno Y, Kwon T, Chapman JA, Toyoda A, Takahashi S, Fukui A, Hikosaka A, Suzuki A, Kondo M, van Heeringen SJ, Quigley I, Heinz S, Ogino H, Ochi H, Hellsten U, Lyons JB, Simakov O, Putnam N, Stites J, Kuroki Y, Tanaka T, Michiue T, Watanabe M, Bogdanovic O, Lister R, Georgiou G, Paranjpe SS, van Kruijsbergen I, Shu S, Carlson J, Kinoshita T, Ohta Y, Mawaribuchi S, Jenkins J, Grimwood J, Schmutz J, Mitros T, Mozaffari SV, Suzuki Y, Haramoto Y, Yamamoto TS, Takagi C, Heald R, Miller K, Haudenschild C, Kitzman J, Nakayama T, Izutsu Y, Robert J, Fortriede J, Burns K, Lotay V, Karimi K, Yasuoka Y, Dichmann DS, Flajnik MF, Houston DW, Shendure J, DuPasquier L, Vize PD, Zorn AM, Ito M, Marcotte EM, Wallingford JB, Ito Y, Asashima M, Ueno N, Matsuda Y, Veenstra GJC, Fujiyama A, Harland RM, Taira M, Rokhsar DS (2016). Genome evolution in the allotetraploid frog Xenopus laevis. Nature.

[37] Kalsotra A, Cooper TA (2011). Functional consequences of developmentally regulated alternative splicing. Nat Rev Genet.

[38] Li Q, Lee JA, Black DL (2007). Neuronal regulation of alternative pre-mRNA splicing. Nat Rev Neurosci.

[39] Sebestyen E, Singh B, Minana B, Pages A, Mateo F, Pujana MA, et al. Large-scale analysis of genome and transcriptome alterations in multiple tumors unveils novel cancer-relevant splicing networks. Genome Res. 2016; Epub ahead of print. 10.1101/gr.199935.115 PMC4889968 27197215

[40] Parikshak NN, Swarup V, Belgard TG, Irimia M, Ramaswami G, Gandal MJ, Hartl C, Leppa V, Ubieta LT, Huang J (2016). Genome-wide changes in lncRNA, splicing, and regional gene expression patterns in autism. Nature.

[41] Elorza A, Marquez Y, Cabrera JR, Sanchez-Trincado JL, Santos-Galindo M, Hernandez IH, et al. Huntington’s disease-specific mis-splicing unveils key effector genes and altered splicing factors. Brain. 2021;awab087. 10.1093/brain/awab087 PMC8370404 33725094

[42] Irimia M, Rukov JL, Roy SW, Vinther J, Garcia-Fernandez J (2009). Quantitative regulation of alternative splicing in evolution and development. Bioessays.

[43] Saito Y, Miranda-Rottmann S, Ruggiu M, Park CY, Fak JJ, Zhong R, Duncan JS, Fabella BA, Junge HJ, Chen Z, Araya R, Fritzsch B, Hudspeth AJ, Darnell RB (2016). NOVA2-mediated RNA regulation is required for axonal pathfinding during development. eLife.

[44] Sapiro AL, Freund EC, Restrepo L, Qiao H, Bhate A, Li Q, Ni J, Mosca TJ, Li JB (2020). Zinc finger RNA-binding protein Zn72D regulates ADAR-mediated RNA editing in neurons. Cell Rep.

[45] Brooks AN, Yang L, Duff MO, Hansen KD, Park JW, Dudoit S, Brenner SE, Graveley BR (2011). Conservation of an RNA regulatory map between Drosophila and mammals. Genome Res.

[46] Irimia M, Denuc A, Burguera D, Somorjai I, Martín-Durán JM, Genikhovich G, Jimenez-Delgado S, Technau U, Roy SW, Marfany G, Garcia-Fernàndez J (2011). Stepwise assembly of the nova-regulated alternative splicing network in the vertebrate brain. Proc Natl Acad Sci U S A.

[47] Solana J, Irimia M, Ayoub S, Orejuela MR, Zywitza V, Jens M, Tapial J, Ray D, Morris Q, Hughes TR, Blencowe BJ, Rajewsky N (2016). Conserved functional antagonism of CELF and MBNL proteins controls stem cell-specific alternative splicing in planarians. eLife.

[48] Burguera D, Marquez Y, Racioppi C, Permanyer J, Torres-Mendez A, Esposito R, Albuixech-Crespo B, Fanlo L, D’Agostino Y, Gohr A (2017). Evolutionary recruitment of flexible Esrp-dependent splicing programs into diverse embryonic morphogenetic processes. Nat Commun.

[49] Ule J, Ule A, Spencer J, Williams A, Hu JS, Cline M, Wang H, Clark T, Fraser C, Ruggiu M, Zeeberg BR, Kane D, Weinstein JN, Blume J, Darnell RB (2005). Nova regulates brain-specific splicing to shape the synapse. Nat Genet.

[50] Seshaiah P, Miller B, Myat MM, Andrew DJ (2001). pasilla, the Drosophila homologue of the human Nova-1 and Nova-2 proteins, is required for normal secretion in the salivary gland. Dev Biol.

[51] Gohr A, Irimia M (2019). Matt: Unix tools for alternative splicing analysis. Bioinformatics.

[52] Ule J, Stefani G, Mele A, Ruggiu M, Wang X, Taneri B, Gaasterland T, Blencowe BJ, Darnell RB (2006). An RNA map predicting Nova-dependent splicing regulation. Nature.

[53] Zhang C, Frias MA, Mele A, Ruggiu M, Eom T, Marney CB, Wang H, Licatalosi DD, Fak JJ, Darnell RB (2010). Integrative modeling defines the nova splicing-regulatory network and its combinatorial controls. Science.

[54] Roy SW, Fedorov A, Gilbert W (2003). Large-scale comparison of intron positions in mammalian genes shows intron loss but no gain. Proc Natl Acad Sci U S A.

[55] Roy SW, Hartl DL (2006). Very little intron loss/gain in Plasmodium: intron loss/gain mutation rates and intron number. Genome Res.

[56] Csuros M, Rogozin IB, Koonin EV (2011). A detailed history of intron-rich eukaryotic ancestors inferred from a global survey of 100 complete genomes. PLoS Comput Biol.

[57] Coulombe-Huntington J, Majewski J (2007). Intron loss and gain in Drosophila. Mol Biol Evol.

[58] Denoeud F, Henriet S, Mungpakdee S, Aury JM, Da Silva C, Brinkmann H, Mikhaleva J, Olsen LC, Jubin C, Cañestro C (2010). Plasticity of animal genome architecture unmasked by rapid evolution of a pelagic tunicate. Science.

[59] Huff JT, Zilberman D, Roy SW (2016). Mechanism for DNA transposons to generate introns on genomic scales. Nature.

[60] Roy SW, Gilbert W (2005). Complex early genes. Proc Natl Acad Sci U S A.

[61] Gelfman S, Burstein D, Penn O, Savchenko A, Amit M, Schwartz S, Pupko T, Ast G (2012). Changes in exon-intron structure during vertebrate evolution affect the splicing pattern of exons. Genome Res.

[62] Alekseyenko AV, Kim N, Lee CJ (2007). Global analysis of exon creation versus loss and the role of alternative splicing in 17 vertebrate genomes. RNA.

[63] Katoh K, Standley DM (2013). MAFFT multiple sequence alignment software version 7: improvements in performance and usability. Mol Biol Evol.

[64] Kumar S, Stecher G, Suleski M, Hedges SB (2017). TimeTree: a resource for timelines, timetrees, and divergence time. Mol Biol Evol.

[65] Pertea G, Pertea M (2020). GFF Utilities: GffRead and GffCompare. F1000Res.

[66] Martín G, Márquez Y, Mantica F, Duque P, Irimia M (2021). Alternative splicing landscapes in Arabidopsis thaliana across tissues and stress conditions highlight major functional differences with animals. Genome Biol.

[67] Ray D, Kazan H, Cook KB, Weirauch MT, Najafabadi HS, Li X, Gueroussov S, Albu M, Zheng H, Yang A, Na H, Irimia M, Matzat LH, Dale RK, Smith SA, Yarosh CA, Kelly SM, Nabet B, Mecenas D, Li W, Laishram RS, Qiao M, Lipshitz HD, Piano F, Corbett AH, Carstens RP, Frey BJ, Anderson RA, Lynch KW, Penalva LOF, Lei EP, Fraser AG, Blencowe BJ, Morris QD, Hughes TR (2013). A compendium of RNA-binding motifs for decoding gene regulation. Nature.

[68] Marquez Y, Mantica F, Cozzuto L, Burguera D, Hermoso-Pulido A, Ponomarenko J, et al. ExOrthist: a tool to infer exon orthologies at any evolutionary distance. Zenodo. 2021. 10.1186/s13059-021-02441-9 PMC8379844 34416914

