# Supplementary material for: ExOrthist: a tool to infer exon orthologies at any evolutionary distance
Source: Genome Biol. 2021 Aug 20;22:239. doi: 10.1186/s13059-021-02441-9 (PMC8379844; doi:10.1186/s13059-021-02441-9)
Supplement: Supplementary file 1 — Additional file 1. Contains Figures S1-S4. [file 13059_2021_2441_MOESM1_ESM.pdf]

A

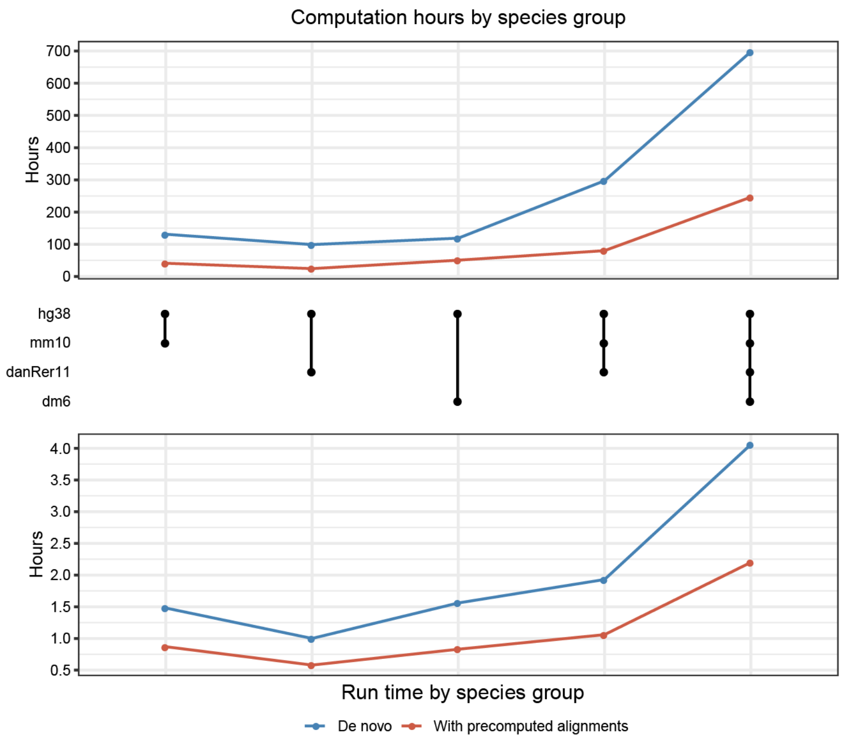

B

Species group	Prev alignment	Evo range: “conservation cut-offs”	CPU hours	Run time
hg38-mm10	No	short: "2,0.50,0.60,0.25"	131.2	1h 29m 9s
hg38-mm10	Yes	short: "2,0.50,0.60,0.25"	41.2	52m 18s
hg38-danRer11	No	medium: "2,0.30,0.60,0.20"	99.1	1h 18s
hg38-danRer11	Yes	medium: "2,0.30,0.60,0.20"	24.5	35m 43s
hg38-dm6	No	long: "2,0.10,0.40,0.15"	119.0	1h 34m 46s
hg38-dm6	Yes	long: "2,0.10,0.40,0.15"	50.7	50m 38s
hg38-mm10-danRer11	No	hg38-mm10 (short): "2,0.50,0.60,0.25"<br>hg38-danRer11 (medium): "2,0.30,0.60,0.20"<br>mm10-danRer11 (medium): "2,0.30,0.60,0.20"	297.3	1h 56m 35s
hg38-mm10-danRer11	Yes	hg38-mm10 (short): "2,0.50,0.60,0.25"<br>hg38-danRer11 (medium): "2,0.30,0.60,0.20"<br>mm10-danRer11 (medium): "2,0.30,0.60,0.20"	80.3	1h 4m 49s
hg38-mm10-danRer11-dm6	No	hg38-mm10 (short): "2,0.50,0.60,0.25"<br>hg38-danRer11 (medium): "2,0.30,0.60,0.20"<br>mm10-danRer11 (medium): "2,0.30,0.60,0.20"<br>hg38-dm6 (long): "2,0.10,0.40,0.15"<br>mm10-dm6 (long): "2,0.10,0.40,0.15"<br>danRer11-dm6 (long): "2,0.10,0.40,0.15"	697.8	4h 4m 31s
hg38-mm10-danRer11-dm6	Yes	hg38-mm10 (short): "2,0.50,0.60,0.25"<br>hg38-danRer11 (medium): "2,0.30,0.60,0.20"<br>mm10-danRer11 (medium): "2,0.30,0.60,0.20"<br>hg38-dm6 (long): "2,0.10,0.40,0.15"<br>mm10-dm6 (long): "2,0.10,0.40,0.15"<br>danRer11-dm6 (long): "2,0.10,0.40,0.15"	246.4	2h 12m 53s

Figure S1 – Computational performance of *ExOrthist*. (a) CPU hours and run time required by each of the pairwise and multi-species runs used to evaluate *ExOrthist* computational performance (see Methods). The species considered in each run are indicated by black dots in the middle section of the plot. The blue line charts refer to *de novo ExOrthist* runs, while the orange line charts refer to runs performed with all pre-computed pairwise alignments (--prevaln option). **(b)** Complete information for the *ExOrthist main* runs depicted in (a): required CPU hours, run time as well as other essential information (species, evolutionary distance ranges, conservation cut-offs, and whether or not the run included

previously generated alignments). The cut-offs are in the format required by *ExOrthist main*: "int_num,ex_seq,ex_len,prot_sim". Additional information can be found in the *Nextflow* report for each of the runs (Additional file 2: Files S1-S10).

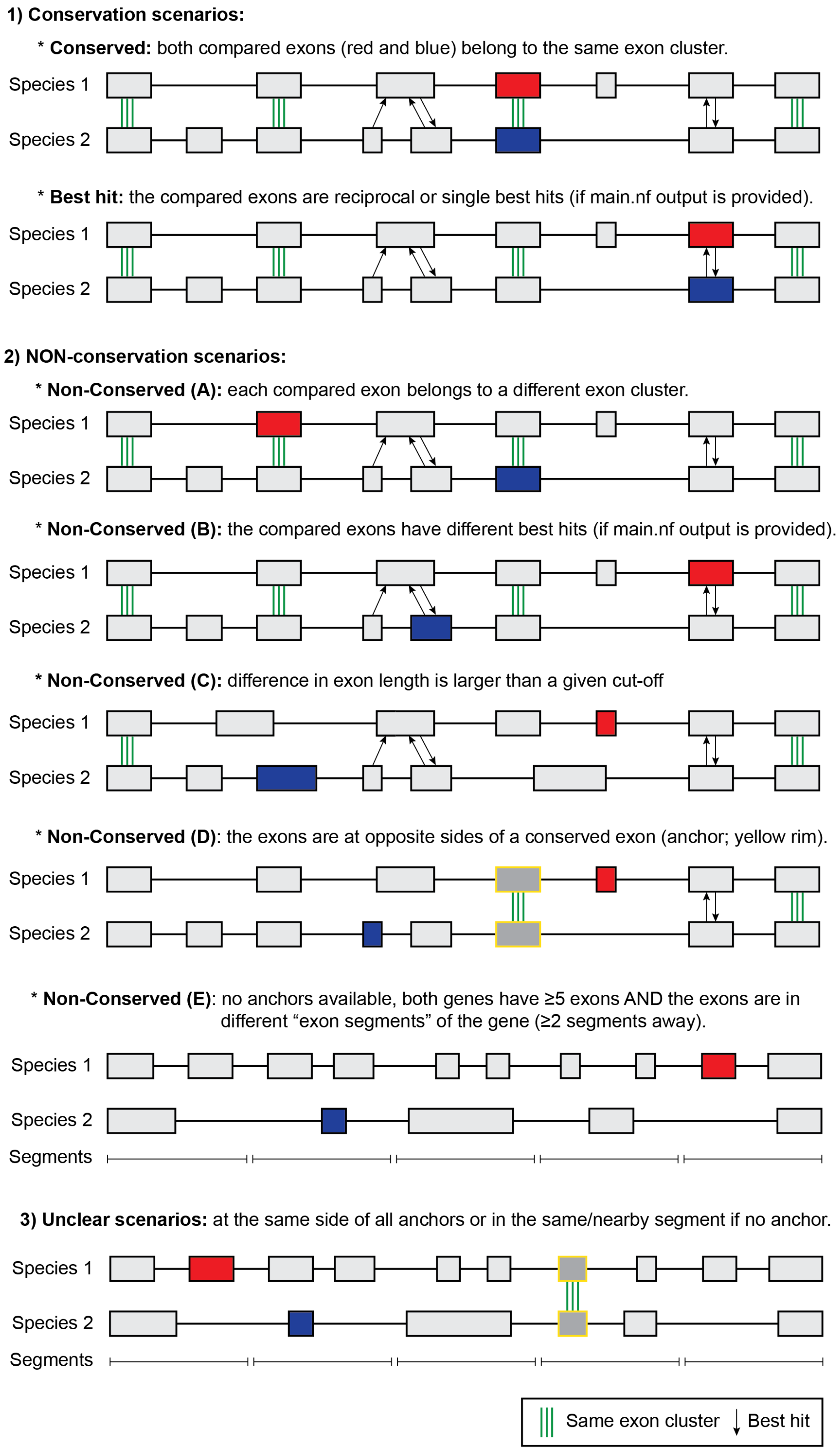

Figure S2 - Scenarios of pairwise exon comparisons in orthologous genes by *compare_exon_sets*. Each non-conservation scenario is evaluated in a consecutive manner (i.e. if the conditions for A are met, the rest of the scenarios are not assessed).

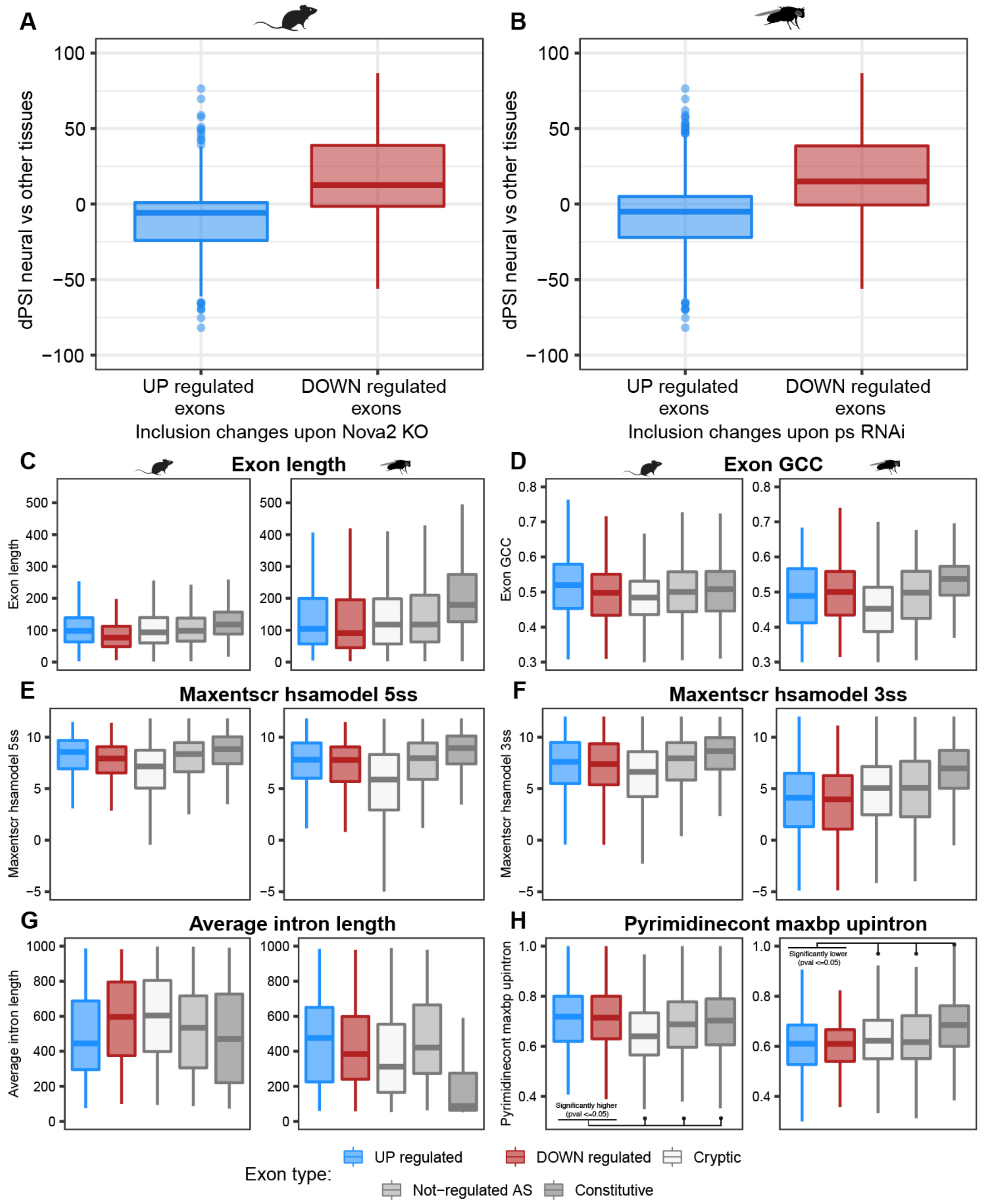

Figure S3 – Features of *Nova*-regulated exons in mouse and fruitfly. (a,b) Distributions of delta PSIs (i.e. differences in inclusion levels) between neural and non-neural samples for exons that are up-regulated (blue) or down-regulated (red) upon *Nova2* depletion in mouse (a) or *ps* knock-down in fruitfly (b). **(c-h)** Comparison of selected exon features as assessed by *matt cmpr_exons* (see Methods). Boxplots in each panel show the distributions of the feature across *Nova*-regulated exons (blue and red; respectively up- and down-regulated upon *Nova* perturbation) and three sets of control exons (cryptic exons, alternative exons not

regulated by *Nova* and constitutive exons) for the mouse (left) or fruitfly (right). Significant differences (Mann-Whitney U tests) between regulated exons and the control sets were only reported for features where the regulated exons presented different trends in the two species. The single panels refer to exon length (c), exon GC content (d), 5′ splice site strength (e), 3′ splice site strength (f), average intron length (between upstream and downstream introns) (g), and length of the pyrimidine tract in the upstream intron (h).

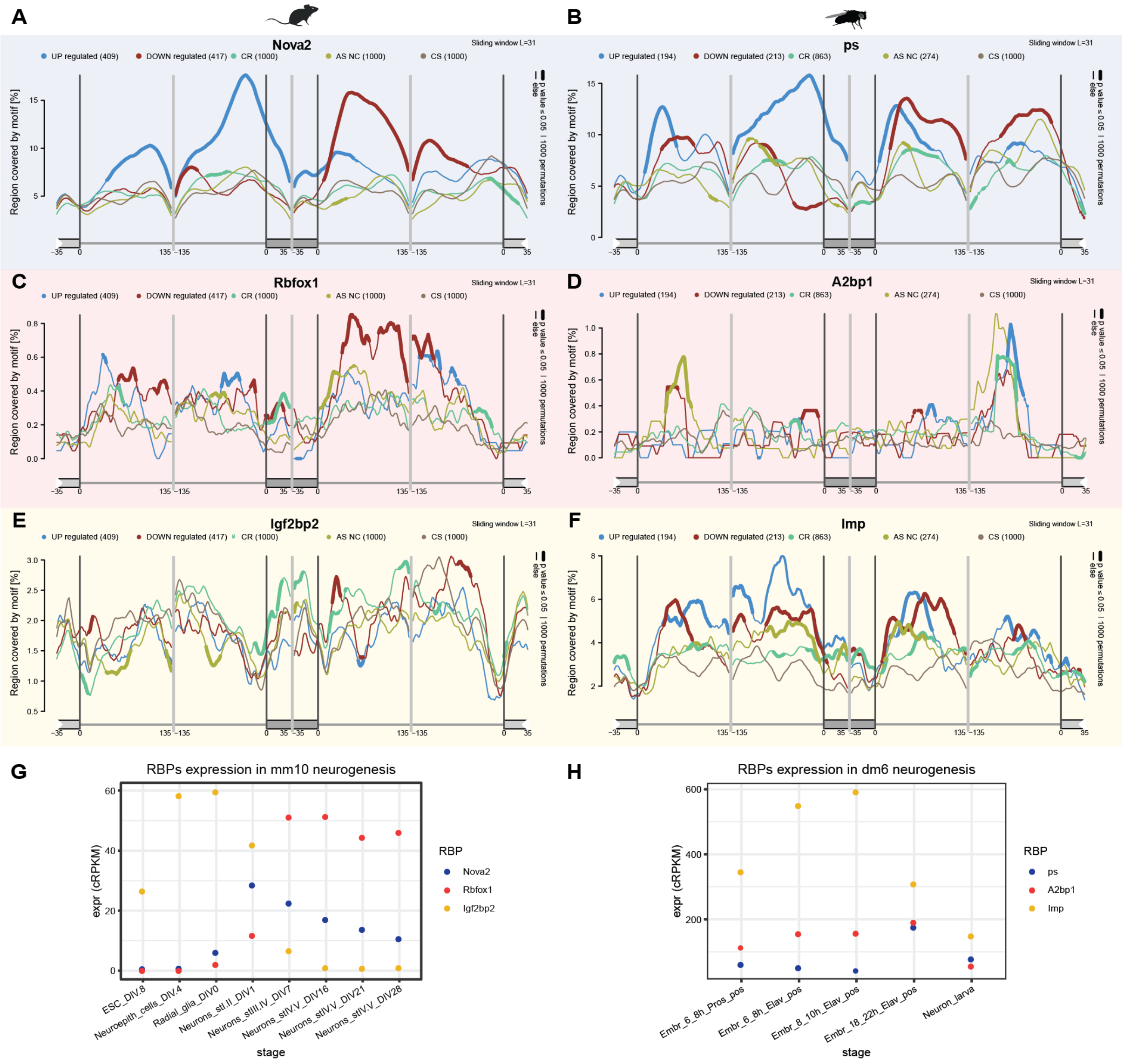

Figure S4 – Motif enrichments around *Nova*-regulated exons. (a-f) RNA maps testing motif enrichment around *Nova*-regulated exons for specific RNA binding proteins (RBPs) in mouse (a,c,e) and their respective orthologs in fruitfly (b,d,f). Thicker lines correspond to significantly enriched regions based on permutations tests done by *Matt*. **(g,h)** Expression patterns of the RBPs represented in (a-f) during mouse neuronal differentiation (g) and fruitfly neurogenesis (h). Expression data obtained from *VastDB*.
