# Supplementary material for: ExOrthist: a tool to infer exon orthologies at any evolutionary distance
Source: Genome Biol. 2021 Aug 20;22:239. doi: 10.1186/s13059-021-02441-9 (PMC8379844; doi:10.1186/s13059-021-02441-9)
Supplement: Supplementary file 4 — Additional file 4. Review history. [file 13059_2021_2441_MOESM4_ESM.docx]

Review History

**First round of review**

**Reviewer 1**

**Were you able to assess all statistics in the manuscript, including the appropriateness of statistical tests used?** No, I do not feel adequately qualified to assess the statistics.

**Were you able to directly test the methods?** No

**Comments to author:**

This manuscript describes the generation and utility of a novel tool (ExOrthist) to determine exon orthogroups and orthopairs across species. It builds on insights gained from similar tools that evaluate evolutionary relationships of genes. The authors focus on analyzing exons as units of investigation, taking into account exon/intron architectures. ExOrthist consists of three modules, one to infer exon homology, one visualizing exon evolution, and one assessing the conservation of alternative splicing. At the heart of the main module that infers exon homology is an approach that not only takes into consideration the sequence and length conservation of the queried exon, but also the conservation of neighboring introns and flanking exons. These added features are based on molecular insights that demonstrate the importance of the immediate surrounding of exons in mediating their inclusion efficiency. The manuscript is divided into the description of ExOrthist and the derivation of appropriate feature cut-offs and an example of application in the context of Nova-mediated alternative splicing. Overall, ExOrthist is a new tool that is likely to be useful for the research community. Having said that, one of the major criticisms of the manuscript is its accessibility to the target audience, which I presume are biologist not necessarily versatile in computer science. The major emphasis is on developing ExOrthist and calibrating cut-offs for various comparisons. Unfortunately, the application of the tool takes little emphasis and should be highlighted more.

At the heart of ExOrthist is a global score that is comprised by the sum of conservation scores of the immediately upstream and downstream intron positions and phases, conservation scores of the query exon sequence and conservation scores of the immediately upstream and downstream exon sequences. It appears that this approach is what makes EXOrthist work efficiently. However, very little information is given about the actual features that make up these categories. How are the immediately upstream and downstream intron positions and phases evaluated, meaning how is a score derived? Sequences, length, etc?, Similarly, how are query exon sequence on length evaluated? What exon length differences are good, not good. This information is important as it likely relies of architectural features that are easily followed and presumably have biological origin.

Along the same line, in the methods the weight of the 5 features is defined. How was the importance for each feature derived? Even more so, if indeed the flanking intron scores are the main contributors in the scoring approach, what biological conclusions can be drawn from these observations?

Can the Novo analysis be extended more to derive novel insights into alternative splicing?

**Reviewer 2**

**Were you able to assess all statistics in the manuscript, including the appropriateness of statistical tests used?** Yes, and I have assessed the statistics in my report.

**Were you able to directly test the methods?** Yes

**Comments to author:**

In this paper the authors present ExOrthist, a software aiming to compute exon level orthology. The author first describes the software and the way they tuned some of its parameters, they then compare its exon orthology prediction with another inference method, and finally presents two use cases for the software : comparing exons orthology between Xenopus laevis and Xenopus tropicalis to study exon evolution after a Whole Genome Duplication and comparing exons regulation of orthologous splicing factors on mouse and fruit fly.

Orthology is the cornerstone of comparative genomics and analysis, and orthology inference at the gene level is currently provided by several inference softwares. ExOrthists is one the firsts software to compute orthology at the exon level, not at the gene level. I could find other tools dedicated to this task either as a recent preprint (Ay et al, 2020), or older published work (Pavesi et al, 2008). I want to bring these references (on foot of this review) to the attention of the authors, since they are not cited in the paper. However, the methodology proposed here is innovative relative to previous work : specifically it takes into account both sequence similarity and intron positions for homology inference. Second, the options are easily tuneable, depending on the distance between species. Third, it allows for multiple species comparisons at once by defining orthologous groups.

Here the method is compared with a liftOver orthology call procedure, using UCSC chain alignment to infer exon orthology. It is not clear from the comparison which method performs better, given the lack of ground truth, nonetheless they appear to broadly agree with their inferences. Moreover, as the authors note, ExOrthist have the benefit to not rely on whole genome alignment, meaning it can in in principle be used for orthology inference between virtually every sufficiently annotated eukaryotic species.

Overall, I am enthusiastic about the software, which I believe will be of broad use to the community: by providing ortholohgous groups at the exons level, it will facilitate all comparative genomics studies aiming to directly study exon evolution. The software is freely available on Github, well documented, easy to install and I was able to complete a test run on my personal computer. . Thus, I recommend this paper for publication. Nevertheless, I have a few concerns, remarks and suggestions :

Intron position and homology

First, it took me a little time to realize "intron position" means the positions of the (removed) introns relative to the coding sequence, as opposed to the positions relative to the gene sequence.

On page 3, l48 the authors state "[...]preservation the two neighboring intron positions,[...]is a requisite for true exon homology". This is admittedly a minor quibble but I don't think this is true. If an intron is inserted in an ancestral exon in one lineage but not in others then the two new exons in species of this lineage will be homologous (orthologous) to the one exon in species of other lineages and not share both intron positions. This case can in fact be detected by the software, since users can choose how many conserved intron positions are necessary for accepting orthology. However, maybe the phrase can be reformulated?

Exon orthogroups and duplication

-Regarding orthogroups : do duplicated exons of the same gene appear in orthogroups ? It is implied but not explicitly stated

Application

"Evaluation of genomic and regulatory conservation of alternatively spliced exon sets'' is one of the applicative section of this paper. The authors look at exons regulated by the Nova splicing factor and assess its conservation between mouse and fruit fly. The authors show that few of the regulated exons are conserved between species but that orthologous genes in those species are regulated by this factor, through non-orthologous exons. This finding is intriguing but I feel like it is insufficiently demonstrated : for example 80 exons in the mouse (on the 827 regulated) are in genes orthologous to genes with regulated exons in the fruit fly, it would help indicating how many would be found merely by chance, or provide statistical significance.

Computation time

Computation time is described for pairs of species, but not with more than two. It appears relevant because computation time likely grows quadratically with the number of isoforms involved, and thus with the number of species. Is it possible to include computation time with more species, at the very least those used for the methods comparisons? It is worth noting that this may be included in the Supplementary Files but I did not find a way to access them.

Data origin

While proper reference of genome assembly used is indicated in the text, most of the actual source of data where the files were downloaded are lacking from the Methods section.

Algorithmic information

No information is given in the manuscript about the algorithm used as a based for pairwise alignment of isoforms sequences. From the code, it appears to use either MAFFT or Clustal. It should be documented in the Methods section. Similar comment for the sequence similarity scoring scheme. (Appears to be based on BLOSUM62)

Visualization module

The software includes a visualization module and shows an example input in Figure 3C. Insufficient description is given in the legends : why are some exons shown with arrows rather than squares?

I would also appreciate that a functional test script would be available for this module as it is for the main software.

---

Additional references :

Ay et al, ExTraMapper: Exon- and Transcript-level mappings for orthologous gene pairs, BioArxiv (https://www.biorxiv.org/content/10.1101/277723v2.full)

Pavesi et al, Exalign: a new method for comparative analysis of exon-intron gene structures, Nucleic Acid Research

(https://doi.org/10.1093/nar/gkn153)

**Authors Response**

**Point-by-point responses to the reviewers’ comments:**

**Reviewer 1:**

This manuscript describes the generation and utility of a novel tool (ExOrthist) to determine exon orthogroups and orthopairs across species. It builds on insights gained from similar tools that evaluate evolutionary relationships of genes. The authors focus on analyzing exons as units of investigation, taking into account exon/intron architectures. ExOrthist consists of three modules, one to infer exon homology, one visualizing exon evolution, and one assessing the conservation of alternative splicing. At the heart of the main module that infers exon homology is an approach that not only takes into consideration the sequence and length conservation of the queried exon, but also the conservation of neighboring introns and flanking exons. These added features are based on molecular insights that demonstrate the importance of the immediate surrounding of exons in mediating their inclusion efficiency. The manuscript is divided into the description of ExOrthist and the derivation of appropriate feature cut-offs and an example of application in the context of Nova-mediated alternative splicing. Overall, ExOrthist is a new tool that is likely to be useful for the research community. Having said that, one of the major criticisms of the manuscript is its accessibility to the target audience, which I presume are biologist not necessarily versatile in computer science. The major emphasis is on developing ExOrthist and calibrating cut-offs for various comparisons. Unfortunately, the application of the tool takes little emphasis and should be highlighted more.

*Response: We thank the Reviewer for his/her positive comments on the tool. We have focused mainly on the description and calibration of the tool since this is a submission as a Software article. However, we certainly appreciate the results we have obtained applying the tool; therefore, as suggested, we have now tried to emphasize these applications and results wherever possible (Abstract, Introduction and Discussion), and also extended the analysis of Nova-regulated alternative splicing networks in the Results section.*

At the heart of ExOrthist is a global score that is comprised by the sum of conservation scores of the immediately upstream and downstream intron positions and phases, conservation scores of the query exon sequence and conservation scores of the immediately upstream and downstream exon sequences. It appears that this approach is what makes EXOrthist work efficiently. However, very little information is given about the actual features that make up these categories. How are the immediately upstream and downstream intron positions and phases evaluated, meaning how is a score derived? Sequences, length, etc?, Similarly, how are query exon sequence on length evaluated? What exon length differences are good, not good. This information is important as it likely relies of architectural features that are easily followed and presumably have biological origin.

*Response: We agree that this information is key to convey, particularly in a Software article. We describe these specific points in detail in the Methods section (e.g. “Parsing of IPA alignments: Intron conservation information”) and further in the README at the Github webpage. Therefore, we believe the Reviewer refers mainly to the main article (Results) section. We have now added additional information on all the points raised (score derivation, features included and not included, etc.) to both the corresponding Results and/or Methods sections. Regarding the question about what exon length differences are good/not good, assessing the optimal exon length ratio for each evolutionary distance range was the goal of the second calibration analysis (Fig. 2d-f). Similarly, the first calibration analysis (Fig. 2a-c) aimed at identifying the optimal minimum exon sequence similarity for the same evolutionary ranges. We have now made this clearer in the text.*

Along the same line, in the methods the weight of the 5 features is defined. How was the importance for each feature derived? Even more so, if indeed the flanking intron scores are the main contributors in the scoring approach, what biological conclusions can be drawn from these observations?

*Response: It is important to clarify here that the global score (given by the sum of the partial scores relative to the 5 features) is only important to select the best representative isoform per gene for each pair of compared exons. Hence, the relative weights are not relevant to decide whether or not two exons are homologs. For two exons to be considered homologs, all tested features must be above their relative cut-offs (number of conserved intron positions, minimum exon sequence similarity, and minimum exon length). We thank the Reviewer for raising this important point that might have caused confusion and we have now made this distinction more explicit in the text (both in the Results and Methods section).*

Can the Novo analysis be extended more to derive novel insights into alternative splicing?

*Response: Thank you for the suggestion. In addition to showing the statistical significance of the overlap between mouse and drosophila orthologous genes hosting nonorthologous Nova-regulated exons both in mouse and fruitfly (i.e. the convergent pattern we observe is not by chance), we have now performed an extensive comparative analysis of the sets of Nova-regulated exons in the two species. In particular, we have investigated their biased neural regulation (previously only assessed for mammals), compared the genomic features associated with each set (including exon and intron lengths, GC contents, splice motif strengths, etc.), and identified enriched motifs for other RNA binding proteins that may indicate coregulation in each lineage. These results are presented in new Supplementary Figures 3 and 4 and described in the following text of the Results section:*

*To gain further insights into the exon programs regulated by Nova in both lineages, we next performed an in-depth comparative analysis. First, we found that exons that were up- and down- regulated upon Nova disruption showed a similar neural depletion and enrichment, respectively, in both mouse and fruitfly (Additional file 1: Figure S3A,B). While Nova-regulated exons are well known to be neurally regulated in mammals [48], this had not been investigated in fruitflies, where ps expression is broader during embryo development [45, 49]. Second, we used matt [50] to evaluate over 50 splicingrelated features in both species. Compared to non-regulated alternative exons and other exon sets in their corresponding species, Nova-regulated exons had overall similar features, including shorter exon lengths (Additional file 1: Figure S3C-G). A notable exception was the length of poly-pyrimidine tract of the upstream intron, which was longer in mouse but shorter than for other exon sets in fruitflies (Additional file 1: Figure S3H). Finally, we also used matt to identify enriched motifs from RNA binding proteins potentially associated with Nova regulation. As expected, Nova-regulated exons from both species exhibited strong enrichment for canonical YCAY clusters in the expected positions based on the direction of regulation (Additional file 1: Figure S4A,B) [44, 51]. Interestingly, we also found enrichment for Rbfox binding motifs downstream of mouse Nova-dependent exons (Additional file 1: Figure S4C), in line with previous reports [52]. However, this enrichment was not observed for flies, suggesting the co-regulation by both factors is lineage-specific (Additional file 1: Figure S4D). Conversely, Nova-regulated exons in Drosophila, but not in mouse, exhibited enrichment for motifs for IGF2BP2/Imp (Additional file 1: Figure S4E,F), an RNA binding protein upregulated during early neuronal differentiation in both lineages (Additional file 1: Figure S4G,H).*

**Reviewer 2:**

In this paper the authors present ExOrthist, a software aiming to compute exon level orthology. The author first describes the software and the way they tuned some of its parameters, they then compare its exon orthology prediction with another inference method, and finally presents two use cases for the software : comparing exons orthology between Xenopus laevis and Xenopus tropicalis to study exon evolution after a Whole Genome Duplication and comparing exons regulation of orthologous splicing factors on mouse and fruit fly.

Orthology is the cornerstone of comparative genomics and analysis, and orthology inference at the gene level is currently provided by several inference softwares. ExOrthists is one the firsts software to compute orthology at the exon level, not at the gene level. I could find other tools dedicated to this task either as a recent preprint (Ay et al, 2020), or older published work (Pavesi et al, 2008). I want to bring these references (on foot of this review) to the attention of the authors, since they are not cited in the paper. However, the methodology proposed here is innovative relative to previous work : specifically it takes into account both sequence similarity and intron positions for homology inference. Second, the options are easily tuneable, depending on the distance between species. Third, it allows for multiple species comparisons at once by defining orthologous groups.

Here the method is compared with a liftOver orthology call procedure, using UCSC chain alignment to infer exon orthology. It is not clear from the comparison which method performs better, given the lack of ground truth, nonetheless they appear to broadly agree with their inferences. Moreover, as the authors note, ExOrthist have the benefit to not rely on whole genome alignment, meaning it can in in principle be used for orthology inference between virtually every sufficiently annotated eukaryotic species.

Overall, I am enthusiastic about the software, which I believe will be of broad use to the community: by providing ortholohgous groups at the exons level, it will facilitate all comparative genomics studies aiming to directly study exon evolution. The software is freely available on Github, well documented, easy to install and I was able to complete a test run on my personal computer. Thus, I recommend this paper for publication.

*Response: We thank the Reviewer for his/her positive comments on the software and for calling our attention to other studies dealing with exon/isoform level conservation. We have now cited them in our manuscript.*

Nevertheless, I have a few concerns, remarks and suggestions :

Intron position and homology

First, it took me a little time to realize "intron position" means the positions of the (removed) introns relative to the coding sequence, as opposed to the positions relative to the gene sequence.

*Response: Thank you for pointing this out. We have now made this more explicit in the text (Results, Methods and the legend of Figure 1).*

On page 3, l48 the authors state "[...]preservation the two neighboring intron positions,[...]is a requisite for true exon homology". This is admittedly a minor quibble but I don't think this is true. If an intron is inserted in an ancestral exon in one lineage but not in others then the two new exons in species of this lineage will be homologous (orthologous) to the one exon in species of other lineages and not share both intron positions. This case can in fact be detected by the software, since users can choose how many conserved intron positions are necessary for accepting orthology. However, maybe the phrase can be reformulated?

*Response: We certainly see the Reviewer's point here and, as indicated, this is why the number of required conserved intron positions can be set to 2, 1 and 0, although in this manuscript we have always used int_num = 2. This is probably more a semantic than a conceptual discussion, but in our opinion “full” (perhaps better than “true”) homology would only apply to those cases in which the pairs of exons are inferred to be ancestrally the “same” entity; therefore, if a new intron is inserted within an exon, this event redefines the ancestral exon into two new exons, which are, individually, no longer fully homologous to the ancestral one. We have now modified the sentence to try to capture the Reviewer’s comment.*

Exon orthogroups and duplication

-Regarding orthogroups : do duplicated exons of the same gene appear in orthogroups ? It is implied but not explicitly stated.

*Response: Yes, they do. We had mentioned this in Results (page 5). We have now made this more explicit and also added the information in the Methods section.*

Application

"Evaluation of genomic and regulatory conservation of alternatively spliced exon sets'' is one of the applicative section of this paper. The authors look at exons regulated by the Nova splicing factor and assess its conservation between mouse and fruit fly. The authors show that few of the regulated exons are conserved between species but that orthologous genes in those species are regulated by this factor, through non-orthologous exons. This finding is intriguing but I feel like it is insufficiently demonstrated : for example 80 exons in the mouse (on the 827 regulated) are in genes orthologous to genes with regulated exons in the fruit fly, it would help indicating how many would be found merely by chance, or provide statistical significance.

*Response: We have now tested the significance of the overlap between gene orthogroups with Nova-regulated exons using a hypergeometric test (P = 1.7e-13). We have also expanded the comparative analysis between the sets of Nova-regulated exons in both lineages to more comprehensively characterize them and to provide further regulatory insights, as suggested by Reviewer 1 (Supplementary Figures 3 and 4).*

Computation time

Computation time is described for pairs of species, but not with more than two. It appears relevant because computation time likely grows quadratically with the number of isoforms involved, and thus with the number of species. Is it possible to include computation time with more species, at the very least those used for the methods comparisons? It is worth noting that this may be included in the Supplementary Files but I did not find a way to access them.

*Response: First, we would like to apologize for the difficulties accessing the Supplementary Files. These were also provided at the end of the merged PDF, but we realize it was not clear. We have now added the statistics on computational performance for runs including three and four species. Since each pair of species is compared, the run time will indeed increase to some extent exponentially (only for the alignment steps). It should be noted, however, that ExOrthist allows the user to provide pre-computed alignments, which means that, to add a new species to a group of already-run species, only the pairwise comparisons with the new species will need to be done. Similarly, if a user wants to explore different cut-offs, this can also be done quickly using the precomputed alignments. Finally, to facilitate the use of ExOrthist for researchers, we have now uploaded pre-computed alignments generated with the latest ExOrthist version for human, mouse, zebrafish and fly (e.g. https://vastdb.crg.eu/IPA_alns/hg38- mm10.tar.gz), and we expect to upload more species pairs in the near future.*

Data origin

While proper reference of genome assembly used is indicated in the text, most of the actual source of data where the files were downloaded are lacking from the Methods section.

*Response: We have added this information in the new Methods section “Genome annotation and sequence files”.*

Algorithmic information

No information is given in the manuscript about the algorithm used as a based for pairwise alignment of isoforms sequences. From the code, it appears to use either MAFFT or Clustal. It should be documented in the Methods section. Similar comment for the sequence similarity scoring scheme. (Appears to be based on BLOSUM62)

*Response: We have added this information, thank you. It indeed uses MAFFT and BLOSUM62.*

Visualization module

The software includes a visualization module and shows an example input in Figure 3C. Insufficient description is given in the legends : why are some exons shown with arrows rather than squares?

I would also appreciate that a functional test script would be available for this module as it is for the main software.

*Response: We added more information in the plot itself, as well as the legend. We also provide a test dataset on the Github webpage. Thank you very much for these and the other suggestions.*

**Second round of review**

**Reviewer 1**

The authors have returned a revised manuscript that addresses all reviewer comments.

**Reviewer 2**

I was a reviewer (#2) for the first submission of this manuscript. With the new submission, the author rigorously addressed all my comments and I am satisfied with most answers and changes made to the manuscript. In response to another reviewer, the authors expanded their analysis on NOVA regulated exons; overall I find these additions worthwhile and scientifically sane . I was, again, able to easily install and run the updated code on the test datasets provided by the authors. Thus, I support publication of the manuscript in this state. However, since I had seemingly no access to the updated legends for the main text’s figures, I would advise the editors to assess them a last time before publication.

For completeness sake, below are additional comments on the answer and the change made by the authors in response to my previous comments.

(A: authors’ responses, R: my comments)

A: Thank you for pointing this out. We have now made this more explicit in the text (Results, Methods and the legend of Figure 1).

R: I am satisfied with the modifications the authors made, which I feel make the sentences clearer. I’m guessing (with respect to the protein-coding sequence)’, l7,p4 should read ‘with respect to’ or ‘in respect to’?

With the latest version of the submission, I do not have access to the Figures legends.

A: We certainly see the Reviewer's point here and, as indicated, this is why the number of required conserved intron positions can be set to 2, 1 and 0, although in this manuscript we have always used int_num = 2. This is probably more a semantic than a conceptual discussion, but in our opinion “full” (perhaps better than “true”) homology would only apply to those cases in which the pairs of exons are inferred to be ancestrally the “same” entity; therefore, if a new intron is inserted within an exon, this event redefines the ancestral exon into two new exons, which are, individually, no longer fully homologous to the ancestral one. We have now modified the sentence to try to capture the Reviewer’s comment.

R: I agree that ‘full’ homology between the two exons better describes what the authors mean. The authors are right to say that is only a semantic discussion and I am satisfied with the minor changes to the text.

A: We have now tested the significance of the overlap between gene orthogroups with Nova-regulated exons using a hypergeometric test (P = 1.7e-13). We have also expanded the comparative analysis between the sets of Nova-regulated exons in both lineages to more comprehensively characterize them and to provide further regulatory insights, as suggested by Reviewer 1 (Supplementary Figures 3 and 4).

A: I am satisfied by the change the authors made, including the statistical test and additional numerical characterization of the data in the “Methods” section of the manuscript.

A: First, we would like to apologize for the difficulties accessing the Supplementary Files. These were also provided at the end of the merged PDF, but we realize it was not clear. We have now added the statistics on computational performance for runs including three and four species. Since each pair of species is compared, the run time will indeed increase to some extent exponentially (only for the alignment steps). It should be noted, however, that ExOrthist allows the user to provide pre-computed alignments, which means that, to add a new species to a group of already-run species, only the pairwise comparisons with the new species will need to be done. Similarly, if a user wants to explore different cut-offs, this can also be done quickly using the precomputed alignments. Finally, to facilitate the use of ExOrthist for researchers, we have now uploaded pre-computed alignments generated with the latest ExOrthist version for human, mouse, zebrafish and fly (e.g. https://vastdb.crg.eu/IPA_alns/hg38- mm10.tar.gz), and we expect to upload more species pairs in the near future.

R : I am satisfied with the answer and the addition of a more precise analysis of computation time.The possibility to use precomputed data is a clear advantage, even though considering dozens of species at a time might be a challenge.

A: We added more information in the plot itself, as well as the legend. We also provide a test dataset on the Github webpage. Thank you very much for these and the other suggestions.

R: The addition to the figure helps understanding the figure better. However, I want to point out that I do not have access to the Figures’ legends in this version of the manuscript, thus I can not check either the legend for Figure 1 or Figure 3c.

I appreciate the addition of a test dataset for the visualization module, I was able to run it for a few examples.
